# Genome-wide identification and abiotic stress-responsive pattern of heat shock transcription factor family in *Triticum aestivum* L.

**DOI:** 10.1186/s12864-019-5617-1

**Published:** 2019-04-01

**Authors:** Shuonan Duan, Binhui Liu, Yuanyuan Zhang, Guoliang Li, Xiulin Guo

**Affiliations:** 1 Institute of Genetics and Physiology, Hebei Academy of Agriculture and Forestry Sciences/Plant Genetic Engineering Center of Hebei Province, Shijiazhuang, 050051 China; 2 Institute of Dryland Farming, Hebei Academy of Agriculture and Forestry Sciences, Hengshui, 053000 China; 3 College of Life Science, Hebei Normal University, Shijiazhuang, 050024 China

**Keywords:** Genome-wide, Heat shock factor, *Triticum aestivum*, Expression profile, Abiotic stress

## Abstract

**Background:**

Enhancement of crop productivity under various abiotic stresses is a major objective of agronomic research. Wheat (*Triticum aestivum* L.) as one of the world’s staple crops is highly sensitive to heat stress, which can adversely affect both yield and quality. Plant heat shock factors (Hsfs) play a crucial role in abiotic and biotic stress response and conferring stress tolerance. Thus, multifunctional Hsfs may be potentially targets in generating novel strains that have the ability to survive environments that feature a combination of stresses.

**Result:**

In this study, using the released genome sequence of wheat and the novel Hsf protein HMM (Hidden Markov Model) model constructed with the Hsf protein sequence of model monocot (*Oryza sativa*) and dicot (*Arabidopsis thaliana*) plants, genome-wide *TaHsfs* identification was performed. Eighty-two non-redundant and full-length *TaHsfs* were randomly located on 21 chromosomes. The structural characteristics and phylogenetic analysis with *Arabidopsis thaliana*, *Oryza sativa* and *Zea mays* were used to classify these genes into three major classes and further into 13 subclasses. A novel subclass, TaHsfC3 was found which had not been documented in wheat or other plants, and did not show any orthologous genes in *A. thaliana*, *O. sativa*, or *Z. mays* Hsf families. The observation of a high proportion of homeologous TaHsf gene groups suggests that the allopolyploid process, which occurred after the fusion of genomes, contributed to the expansion of the TaHsf family. Furthermore, *TaHsfs* expression profiling by RNA-seq revealed that the *TaHsfs* could be responsive not only to abiotic stresses but also to phytohormones. Additionally, the TaHsf family genes exhibited class-, subclass- and organ-specific expression patterns in response to various treatments.

**Conclusions:**

A comprehensive analysis of Hsf genes was performed in wheat, which is useful for better understanding one of the most complex Hsf gene families. Variations in the expression patterns under different abiotic stress and phytohormone treatments provide clues for further analysis of the TaHsfs functions and corresponding signal transduction pathways in wheat.

**Electronic supplementary material:**

The online version of this article (10.1186/s12864-019-5617-1) contains supplementary material, which is available to authorized users.

## Background

Wheat (*Triticum aestivum* L.) is a temperate cereal crop that often encounters heat stress during the reproductive stage in warm-climate wheat production regions Heat stress has a substantial adverse impact on carbon assimilation and starch synthesis, resulting in the reduction of grain yield and quality. Wheat is one of the world’s staple crops, and the most crucial target of wheat breeding is high and stable yield and quality. Wheat is a cool season crop having an optimal daytime growing temperature during its reproductive development of 15 °C, and for every degree Celsius above this optimum temperature a reduction of 3–4% in the yield has been observed [1]. Additionally, it is reported that the average global temperature is increasing at a rate of 0.18 °C every decade [2], and starch accumulation in wheat grains decreases by > 30% at temperatures between 30 °C and 40 °C [3]. Therefore, the likely impact of heat stress on wheat and the genetic improvement of heat tolerance and its underlying mechanisms have been extensively investigated in recent years.

As sessile organisms, plants could not escape from harmful environments by changing sites, and are exposed to multiple abiotic and biotic stresses frequently [4]. Therefore, a complex stress regulation and response network was developed at biochemical, physiological and molecular levels for stresses adaptation [5, 6]. Many genes which exert a crucial part in this complex stress regulation and response network or confer stress tolerance are mainly regulated by transcription factors [7]. Transcription factors that perform a crucial function in stress signal perception and transduction processes could induce the expression of stress-responsive genes by recognizing and interacting with *cis*-acting elements in their promoter region, thereby the stress tolerance of plants is enhanced by activated stress signal cascade and whole downstream functional genes of this network [8]. Therefore, transcription factors are considered as potent candidates for developing the next-generation transgenic crops with strong stress tolerance.

Among plant transcription factors, Hsfs have recently attracted particular interest because as terminal components of signal transduction chains, plant Hsfs can regulate the expression of genes involved in various abiotic stress responses [9]. Most types of abiotic stresses disrupt the metabolic balance of cells, resulting in an increase in the production of reactive oxygen species (ROS) [10], and the general concept of HS (heat shock) signaling activation is mainly centered on the disruption of cytosolic protein homeostasis and depletion of the pool of free chaperones [11]. Hsfs and their products protect cells from extreme proteotoxic damage via the expression of molecular chaperones such as heat shock proteins (Hsps). Furthermore, it has been proven that Hsfs participate in some stress-related phytohormone signaling pathways such as abscisic acid (ABA) and salicylic acid (SA) [12, 13].

The plant Hsf gene was firstly cloned from tomato in 1990 [14]. The structure analyses revealed that a modular Hsf contains five conserved domains, including two indispensable domains DNA-binding domain (DBD) and oligomerization domain (OD) comprising central helix-turn-helix (HTH) motif and hydrophobic heptad repeats (HR-A and HR-B) respectively in N-terminal, beside three typical domains nuclear localization signal (NLS), nuclear export signal (NES) and activator peptide motif (AHA) are presented in C-terminal of Hsfs [11, 14]. Based on the number of amino acid residues inserted between HR-A and HR-B, plants Hsf can be grouped into three major classes, namely: A, B, and C. There are 21 and 7 amino acid residues insertion between the HR-A and HR-B region of HsfAs and HsfCs respectively. Compared with HsfAs and HsfCs, HsfBs have a shorter HR-A/B without any amino acid residue insertion [9, 15]. Furthermore, the AHA activation domains are identified in HsfAs uniquely which are absent in HsfBs and HsfCs [16]. HsfBs are characterized by the tetrapeptide-LFGV-, which is located within the C-terminal and is predicted to be a repressor motif based on its observed activity in other plant transcription factors [17, 18]. Based on phylogenetic comparisons with the Hsf family of model species such as *Arabidopsis* and rice, plant Hsf family members could further be divided into several subclasses. The specific function of plant Hsf subclasses in model plants also been reported in previous work. *AtHsfA1s* have been proved to be the master regulators of heat stress response in *Arabidopsis*, which could induce the expression of diverse transcription regulators, including other Hsf subclasses (A2, A3, A7, B1 and B2) [19, 20]*. AtHsfA2* not only confers heat and osmotic stress tolerance, but also plays a significant role in the growth and development of plants [21–23]. The tomato (*Lycopersicon esculentum*) HsfA4s are potent activators of heat stress gene expression, whereas HsfA5s act as specific repressors of HsfA4s activity [24]. The function of OsHsfB2b is considered as a negative regulator in response to drought and salt stresses in rice [25]. *OsHsfC1b* is induced by salt, mannitol and ABA, but not by H_2_O_2_, and play an important role in salt and osmotic stress response [26]. The above results reveal that plants have evolved both subclass-specific and multiple functions in some members of the Hsf family.

The identification of plant Hsf family genes is usually based on the characteristics of the two conserved domains, DBD and HR-A/B, which is the core of the Hsf HMM model, as a query in searching the proteome [27]. Unlike the simple and small Hsf family in animals and yeast, plants have relatively complex and large Hsf gene families. Bread wheat (*T. aestivum*) has one of the most complex genomes known to science, with an overall size of more than 17 billion bases [28]. Additionally, the tetraploid emmer wheat (*T. turgidum*; BBAA) and hexaploid bread wheat (BBAADD) originated within the past few hundred thousand years and ten thousand years ago [29, 30], respectively. It can be predicted that wheat that underwent two rounds of whole genome duplications in recent years must have one of the most complex and largest Hsf family in plants. The recently available *T. aestivum* genome TGACv1 has allowed the identification of *TaHsfs* at the genome-wide level [31].

As a major cereal crop, wheat is widely cultivated around the world, following maize in grain, and provides carbohydrates and proteins for approximately 40% of the world’s population [32]. Wheat is hypersensitive to heat stress, particularly at the early grain filling and reproductive stages, significantly limiting wheat production [33]. This study aimed to elucidate the abiotic stress-responsive pattern of *TaHsfs* and identify candidates for genetic improvement of abiotic tolerance in this species.

## Results

### Identification of Hsf genes in *T. aestivum*

**Fig. 1:**
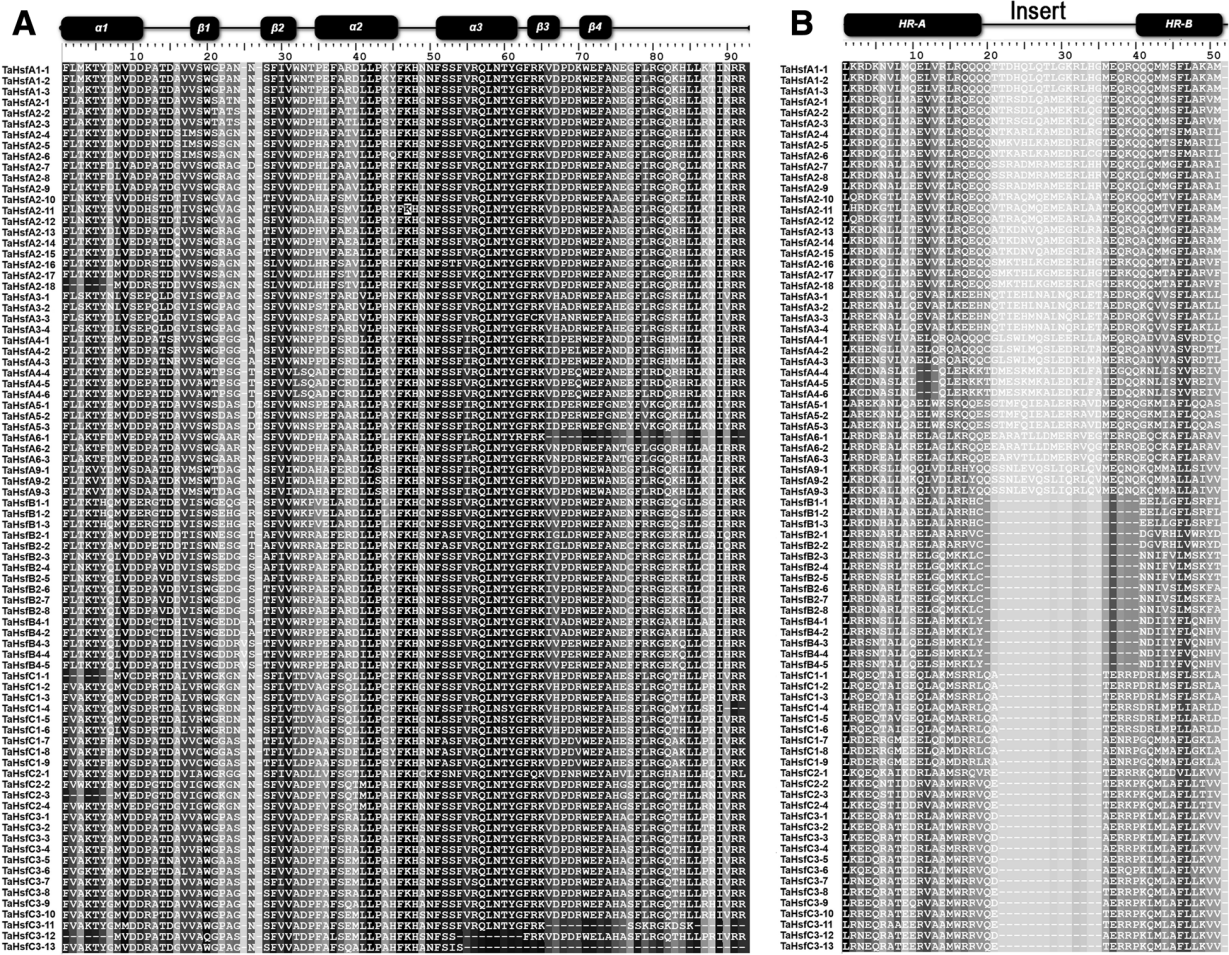
Multiple sequence alignment of the DNA-binding and HR-A/B domains of wheat Hsfs. The protein sequence alignment was performed using the BioEdit software. **a** Multiple alignment clearly reveals that the DBD domains of the wheat Hsfs are highly conserved. The secondary structure elements of the DBD (α1-β1-β2-α2-α3-β3-β4) are shown above the alignment. **b** The scheme at the top shows the boundaries and locations of the HR-A, insert and HR-B regions within the HR-A/B domains. The structures between HR-A and HR-B consist of 21 and 7 amino acid insertions in the TaHsfAs and TaHsfCs, respectively

The constructed HMM for Hsf was based on the protein sequence of *A. thaliana* and *Oryza sativa*, which was queried in BLASTP searches for possible homologous TaHsfs in the *T. aestivum* proteome. A total of 154 candidate Hsf protein sequences were identified in this process. Subsequently, the putative wheat Hsf protein sequences were surveyed to further determine whether these included a DBD and HR-A/B domain using SMART software (http://smart.embl-heidelberg.de/). Consequently, 74 of the candidate Hsf protein sequences were excluded from further analysis based on the absence of DBD or HR-A/B domains and overlapping genes. Eighty nonredundant wheat Hsfs with the DBD and HR-A/B domains were identified and characterized. Additionally, two members of subclass TaHsfA2 (*TaHsfA2–11* and *TaHsfA2–18*) were obtained by homologous cloning (Fig. 1, Additional file 1). The deduced protein sizes ranged from 209 amino acids (TaHsfB2–2) to 701 amino acids (TaHsfB2–7) (Table 1). Based on the number of amino acid residues inserted into the HR-A/B domain, 82 TaHsfs were classified into three major classes; class A contained the highest number of TaHsf members (40), classes B and C consisted of 16 and 26 TaHsf members, respectively (Fig. 1B).

**Table 1:** Details of various TaHsfs, including chromosome locations, transcript ID, protein sequence length (number of amino acids, AA), molecular weight (MW), nuclear export signal (NES), nuclear localization signal (NLS), activator motifs (AHA) and endoplasmic reticulum (ER) membrane retention signals

Gene name	Chromosome	Transcript ID	No. of AA	MW (kDa)	NES	NLS	AHA Motif	ER Membrane Retention Signals
TaHsfA1–1	4A	AA0958570	521	57.36		(126)RRKP(252)KKRR	(464)DSFWEQFLCA	
TaHsfA1–2	5B	AA1285550	529	58.19		(129)RRKP(256)KKRR	(472)DSFWEQFLCA	
TaHsfA1–3	5D	AA1410370	522	57.46		(124)RRKP(249)KKRR	(465)DSFWEQFLCA	
TaHsfA2–1	5A	AA1215960	346	38.99	(157)LKRDRQLLM	(125)KRRKP(223)RKELEDAISNKRRRRID	(313)DDFWEDLLHE	(2)SHRM(342)AEKL
TaHsfA2–2	5B	AA2081150	353	39.73	(164)LKRDRQLLM	(132)KRRKP(230)RKELEDAISNKRRRRID	(320)DDFWEDLLHE	(2)SHRM(349)AQKM
TaHsfA2–3	5D	AA1442920	348	38.91	(159)LKRDRQLLM	(127)KRRKP(225)RKELEDAISNKRRRRID	(315)DDFWEDLLHE	(344)AEKL
TaHsfA2–4	2A	AA0357000	413	45.61		(130)RRRKP(229)RKELHDAISKKRRRRID	(370)DNFWEELLNK	
TaHsfA2–5	2B	AA0492290	405	44.93		(131)RRRKP(239)KKRRRR	(362)DNFWEGLLNK	
TaHsfA2–6	2D	AA0573260	412	45.44		(130)RRRKP(229)RKELHDAISKKRRRRID	(369)DNFWEELLNK	
TaHsfA2–7	4A	AA1018260	341	39.61		(133)KRRRP(241)PTKRRRP	(311)DDFWEELLSE	(2)DRVL
TaHsfA2–8	4B	AA1062420	341	39.6		(133)KRRR(241)PTKRRRP	(311)DDFWEELLSE	(2)DRVL
TaHsfA2–9	4D	AA1126990	341	39.5		(133)KRRRP(243)KRRR	(311)DDFWEELLSE	
TaHsfA2–10	5A	AA1256510	372	41.14	(320)LLSLGLE	(153)RRRR(259)RRKELAEALLSKKRGRP	(314)ESFWKELLSL	
TaHsfA2–11	U		373	41.44	(322)LLSLGLE	(153)RRRRP(261)RRKELADALLSKKRGRP	(314)ESFWKELLSL	
TaHsfA2–12	5D	AA1405180	377	41.6	(326)LLSLGLE	(153)RRRRP(261)RRKELAEALLSKKRGRP	(320)ESFWKELLSL	
TaHsfA2–13	1A	AA0048030	364	41.03	(166)IDRLKRDKNLLI	(135)KRRKP(234)KRKELEDAISKKRRRPI	(318)NDFWAELFSD	
TaHsfA2–14	1B	AA0114190	364	41.03	(166)IDRLKRDKNLLI	(135)KRRKP(234)KRKELEDAISKKRRRPI	(318)NDFWAELFSD	
TaHsfA2–15	1D	AA0199110	370	42.05	(172)IDRLKRDKNLLI	(141)KRRKP(240)KRKELEDAISKKRRRPI	(324)NDFWAELFSD	
TaHsfA2–16	3A	AA0636800	397	44.24		(129)RRRRP(237)KKRRR	(354)DDFWEELMSR	
TaHsfA2–17	3B	AA0745290	433	48.01		(128)RRRRP(236)KKRRR	(353)DDFWEELMSR	
TaHsfA2–18	3D		407	46.23	(369)LDVYKLDL	(81)RRRRP(189)KKRRR(353)RRRH	(306)DDFWEELMSR	
TaHsfA3–1	2A	AA0373860	467	51.63	(438)FDALDDGDLHL		(402)DTFFQSSCSG	(463)GNMK
TaHsfA3–2	2A	AA0367680	502	55.45			(437)DTFFQSSCSG	(498)GNMK
TaHsfA3–3	2B	AA0455720	475	52.09	(29)LEPKLEM		(433)DTFFQSSCSG	
TaHsfA3–4	2D	AA0576700	499	54.79	(25)LLLEPKLEM(47)EALDDGDLHL		(434)DTFFQSSCSG	(495)GNMK
TaHsfA4–1	1A	AA0026630	443	49.57	(263)MELALVSM	(112)HRRKP(220)KKRR	(386)DLFWERFLTD	(439)SAQK
TaHsfA4–2	1B	AA0108940	445	49.88	(263)MELALVSM	(110)HRRKP(219)KKRR	(388)DLFWERFLTD	(441)SAQK
TaHsfA4–3	1D	AA0193800	442	49.71	(266)MELALVSM	(110)HRRKP(218)KKRR	(385)DLFWERFLTD	(438)SAQK
TaHsfA4–4	3A	AA0661360	432	48.38	(133)LKCDNASLKL	(100)HRRKP(198)KKRR	(370)DGFWQQFLTE	(428)SAEK
TaHsfA4–5	3B	AA0790580	441	49.47	(133)LKCDNASLKL	(100)HRRKP(198)KKRR	(379)DGFWQQFLTE	(437)SAEK
TaHsfA4–6	3D	AA0847210	433	48.46	(133)LKCDNASLNL	(100)HRRKP(198)KKRR	(370)DGFWQQFLTE	(429)SAEK
TaHsfA5–1	6A	AA1567660	458	49.88	(340)LTL	(109)RRKP(212)HKKRR	(414)DNFWEQFLTE	(454)EQLK
TaHsfA5–2	6B	AA1650270	455	49.93	(340)LTL	(109)RRKP(212)HKKRR	(414)DNFWEQFLTE	(451)EQLK
TaHsfA5–3	6D	AA1745290	458	49.87	(340)LTL	(109)RRKP(212)HKKRR	(414)DNFWEQFLTE	(454)EQLK
TaHsfA6–1	7A	AA1799040	310	33.78		(202)KKKRR	(247)DMIWYELLEE	
TaHsfA6–2	7B	AA1908250	351	37.9		(136)RRRR(250)KKKRR	(299)DMIWYELLEE	
TaHsfA6–3	7D	AA2004680	351	37.98		(136)RRRR(250)KKKRR	(299)DMIWYELLEE	
TaHsfA9–1	4A	AA1009080	383	42.86	(165)LKRDKSLLMQQL	(124)KRKKRP(242)KKRR	(336)MHLWFGEDGE	
TaHsfA9–2	4B	AA1071190	384	42.88	(172)LMKQLVDLRL	(124)KRKKRP(232)RRNNCVYEDGNKKRRFP	(337)MHLWFNEDGE	
TaHsfA9–3	4D	AA1142510	384	42.93	(172)LMKQLVDLRL	(124)KRKKRP(232)RRNNCVYEDGNKKRRFP	(337)MHLWFSEDGE	
TaHsfB1–1	5A	AA1196580	298	32.15	(209)LDVRQLDLRLLM	(114)RRRK(103)RRGEQGLLSGIRRRKAT		
TaHsfB1–2	5B	AA1307960	298	32.29	(211)LDVRQLDLRLLM	(117)RRRK(106)RRGEQSLLSGIRRRKAT		
TaHsfB1–3	5D	AA1418380	298	32.06	(211)LDVRQLDLRLLM	(117)RRRK(106)RRGEQSLLSGIRRRKAT		
TaHsfB2–1	2A	AA0301090	295	31.99	(262)MRTERSDLNVLSL	(89)RKGEKRLLGAIQRRKGS(165)RRENARLARELARARRV		
TaHsfB2–2	2D	AA2166870	209	22.73		(102)RKGEKRLLGAIQRRKGS(178)RRENARLARELARARRV		
TaHsfB2–3	7A	AA1829250	374	40.46		(119)HRRK(108)RRGEKRLLCDIHRRKVT		
TaHsfB2–4	7B	AA1935670	374	40.34		(119)HRRK(108)RRGEKRLLCDIHRRKVT		
TaHsfB2–5	7D	AA2015840	367	39.79		(119)HRRK(108)RRGEKRLLCDIHRRKVT		
TaHsfB2–6	5A	AA0058470	404	42.04		(129)HRRK(118)RRGEKRLLCDIHRRKVV		
TaHsfB2–7	5B	AA1315570	701	73.93	(78)LLPLGISLVI	(252)RRGEKRLLCDIHRRKVV(668)RRRP		
TaHsfB2–8	5D	AA1415650	397	41.11		(129)HRRK(118)RRGEKRLLCDIHRRKVV		
TaHsfB4–1	2A	AA2122890	320	35.26	(269)LELDMDV	(115)RKGAKHLLAEIHRRKSS(299)KKKR		
TaHsfB4–2	2D	AA0597540	320	35.32	(269)LELDMDV	(116)RKGAKHLLAEIHRRKSS(299)KKKR		
TaHsfB4–3	5A	AA1189330	388	41.36	(362)LALENPDLSL	(103)RKGEKQLLCEIHRRKTS		(6)ERCG
TaHsfB4–4	5B	AA1293070	388	41.46	(362)LALENPDLSL	(103)RKGEKQLLCEIHRRKTS		(6)ERCG
TaHsfB4–5	5D	AA1418740	388	41.4	(362)LALENPDLSL	(103)RKGEKQLLCEIHRRKTS		(2)ERCG
TaHsfC1–1	3A	AA0634640	294	32.58		(78)PRIVRRK		
TaHsfC1–2	3B	AA0722380	325	35.73	(10)LGLI	(109)PRIVRRK		
TaHsfC1–3	3D	AA0860000	321	35.39	(10)LGLI	(109)PRIVRRK		
TaHsfC1–4	3A	AA0670400	277	31.16	(175)LQQAAEKKLQRMHL			
TaHsfC1–5	3B	AA0722680	304	33.89		(105)PRIVRRK		
TaHsfC1–6	3D	AA0849120	225	25.63		(105)PRIVRRK		
TaHsfC1–7	3A	AA0614700	236	26.06		(103)PLIVRKK(198)PDKRRRI		
TaHsfC1–8	3B	AA0780810	227	24.69		(103)PLIVRKK(203)PDKRRRI		
TaHsfC1–9	3D	AA0873000	241	26.41		(103)PLIVRKK(203)PDKRRRI		
TaHsfC2–1	5A	AA2086000	268	30	(197)VKRLRLQL	(118)KRKPK(162)RRRK		
TaHsfC2–2	7A	AA1794130	266	28.23	(168)LAFLLTVV	(103)RRGTAVGGGGGGKRKDA(162)RKPK		
TaHsfC2–3	7B	AA1874420	244	26.12	(146)LAFLLTI	(140)RKPK		
TaHsfC2–4	7D	AA2011440	265	28.18	(167)LAFLLTI	(101)RRGTAAAAGGGGGKRKD(161)RKPK		
TaHsfC3–1	4D	AA1147440	276	30.27	(169)LMLAFLL	(107)PRIVRRR(206)KRRRLLLDGEGQVSKKKMR		
TaHsfC3–2	5A	AA1225450	274	30.36	(167)LMLAFLL	(163)RRPK(204)KRPRLLLDGEAQMGKKK		
TaHsfC3–3	4B	AA1034070	257	28.73	(169)LMLAFLL	(105)PRIVRRR(199)PARARRPR		
TaHsfC3–4	3B	AA0791830	274	29.98	(168)LAFLL	(103)PRIVRRR(203)KRPRLLLDGEVQVGKKK		
TaHsfC3–5	3B	AA0777840	230	25.57	(170)LAFLL	(108)PRIVRRR(202)KRPR		
TaHsfC3–6	4B	AA1034080	257	28.5	(207)LLLNGEVQM	(104)PRIVRRR(203)KKPR		
TaHsfC3–7	4B	AA1067170	268	30		(158)RRPK		
TaHsfC3–8	4B	AA1045990	273	29.46	(174)LLKV	(165)RRPK(205)KRPR		
TaHsfC3–9	U	AA1138930	276	29.86	(177)LAFLL	(171)RRPK(211)KRPR		
TaHsfC3–10	5A	AA1241720	273	30.18		(173)RRPK		
TaHsfC3–11	U	AA1138960	248	26.43		(141)RRPK(173)KRPR		
TaHsfC3–12	3B	AA0830650	237	26.13	(133)LAFLL	(127)RRPK(168)KRPR		
TaHsfC3–13	4B	AA1040480	248	26.15		(145)RRPK		

**Fig. 2:**
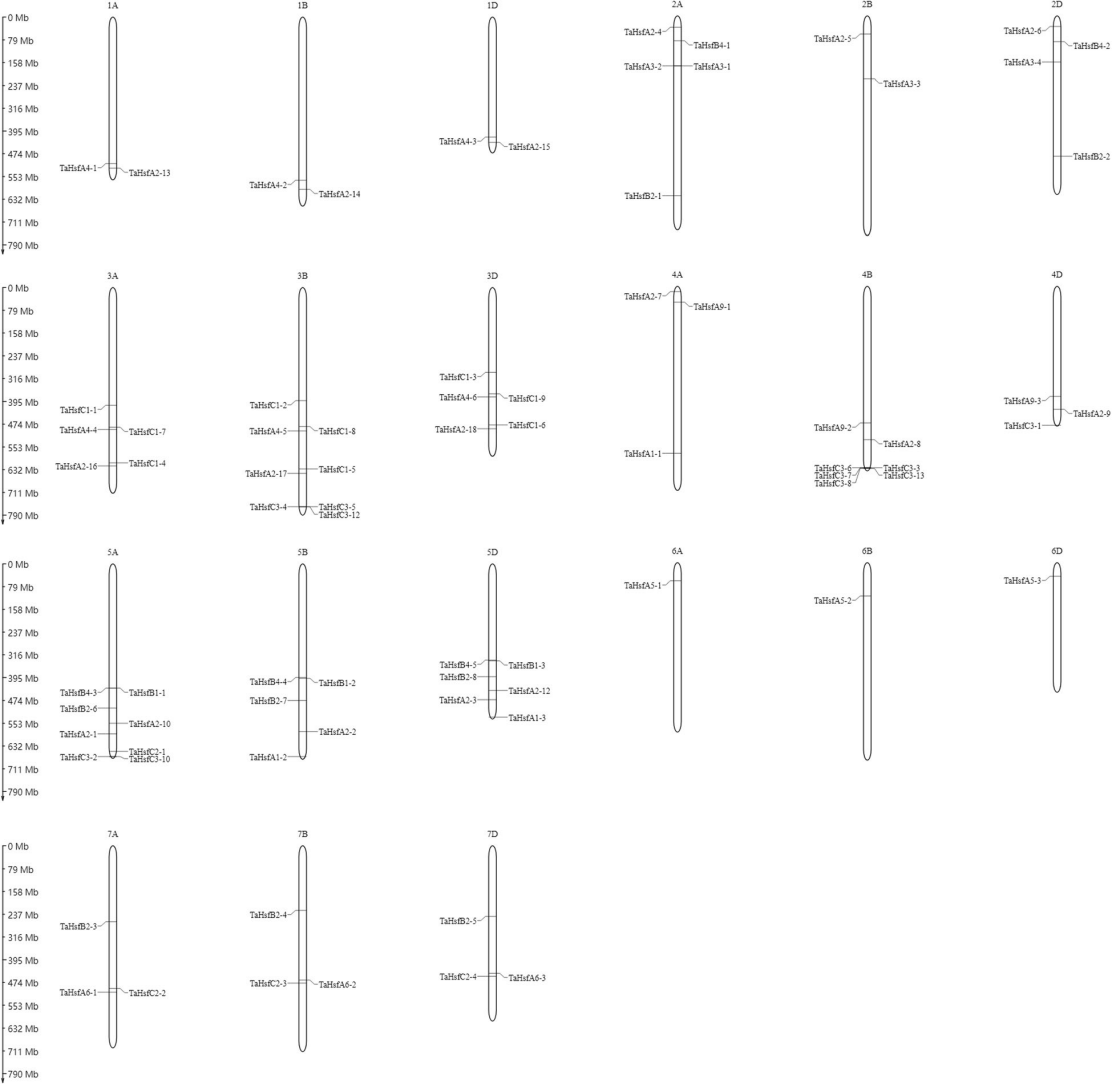
Localization of wheat Hsfs on chromosomes 1A-7D. The scale is represented in megabases (Mb). The chromosome numbers are shown at the top of each bar

Except for three TaHsfs (*TaHsfA2–11*, *TaHsfC3–9* and *TaHsfC3–11*) located on the unanchored scaffolds, 79 nonredundant wheat Hsfs were mapped to 21 wheat chromosomes (Fig. 2, Additional file 2). *TaHsfs* were distributed among 21 wheat chromosomes, but the number of TaHsf genes on each chromosome extensively differed. The highest number of TaHsf genes (*N* = 8) was observed on chromosomes 3B and 5A, whereas the lowest number was detected on chromosomes 6A, 6B and 6D, each of them including only one TaHsf gene. Chromosomes 5 and 3 showed the highest density of TaHsf genes, with 19 and 18 members, respectively. Chromosomes 3A and 3B have previously been reported to likely harbor key genes conferring heat tolerance in wheat [34].

### Phylogenetic analysis and classification of TaHsf members

**Fig. 3:**
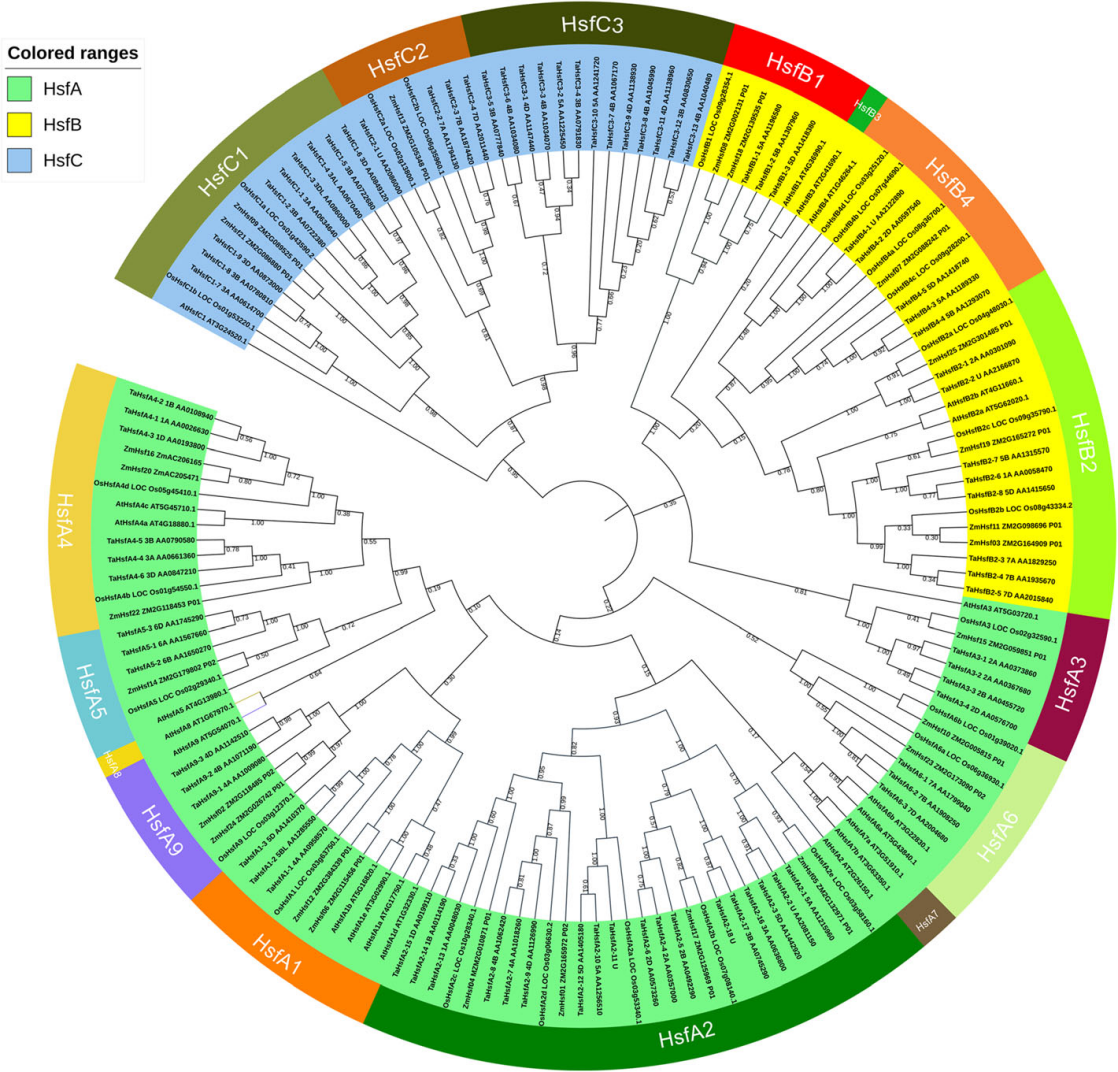
Neighbor-joining phylogenetic tree of wheat, rice, *Arabidopsis* and maize Hsf families. The N-proximal regions (from the start of the DNA-binding domain to the end of the HR-A/B region) of the Hsf proteins were used to construct the phylogenetic tree with MEGA 5.0. For wheat (Ta), rice (Os), Arabidopsis (At) and maize (Zm) Hsf proteins, classes A, B and C are in green, yellow and blue, respectively, both transcript ID and subclass names are shown

To investigate the evolutionary features and characteristics of the TaHsf genes, an unrooted phylogenetic tree was constructed. Phylogenetic analysis was performed based on the Hsfs amino acid sequences of the N-terminal domains of Hsfs, including the DNA-binding domain, the HR-A/B domain and the linker between two domains from *T. aestivum, A. thaliana, O. sativa and Z. mays*. The three major classes, namely, TaHsfA (green), TaHsfB (yellow) and TaHsfC (blue), could also be clearly distinguished by phylogenetic analysis (Fig. 3). The TaHsf family was further divided into 13 subclasses based on their bootstrap values and phylogenetic relationship with the orthologous genes from *O. sativa* and *A. thaliana* (Additional file 3). Class A was further subdivided into seven subclasses, namely, TaHsfA1, TaHsfA2, TaHsfA3, TaHsfA4, TaHsfA5, TaHsfA6 and TaHsfA9. Both classes B and C consisted of three subclasses, which were named as TaHsfB1, TaHsfB2 and TaHsfB4 and TaHsfC1, TaHsfC2 and TaHsfC3, respectively. Although classes A, B and C in both eudicots (*Arabidopsis*) and monocots were conserved, whereas subclasses HsfA2, A6, A9 and B1 were divided into different clade between monocots and dicots. The monocots and dicots uniquely consist of subclasses B4, C2 and A7, A8, B3 respectively. The monocots *T. aestivum, O. sativa* and *Z. mays* followed the same subclassification and were very closely clustered. Subclass A2 had the highest number of Hsfs (*N* = 18) in the wheat Hsf family, whereas the lowest number of Hsfs (*N* = 3) was observed in subclasses A1, A5, A6, A9 and B1. A clade of TaHsfC without orthologs detected in other plants was designated as TaHsfC3. All homeologous TaHsf gene groups with a copy on each of the A, B and D homeologous chromosome were closely clustered. The wheat Hsf genes also exhibited a closer phylogenetic relationship with the monocot rice than with the *Z. mays* and the dicot *Arabidopsis*.

### Gene structure and conserved domains of Hsfs in *T. aestivum*

**Fig. 4:**
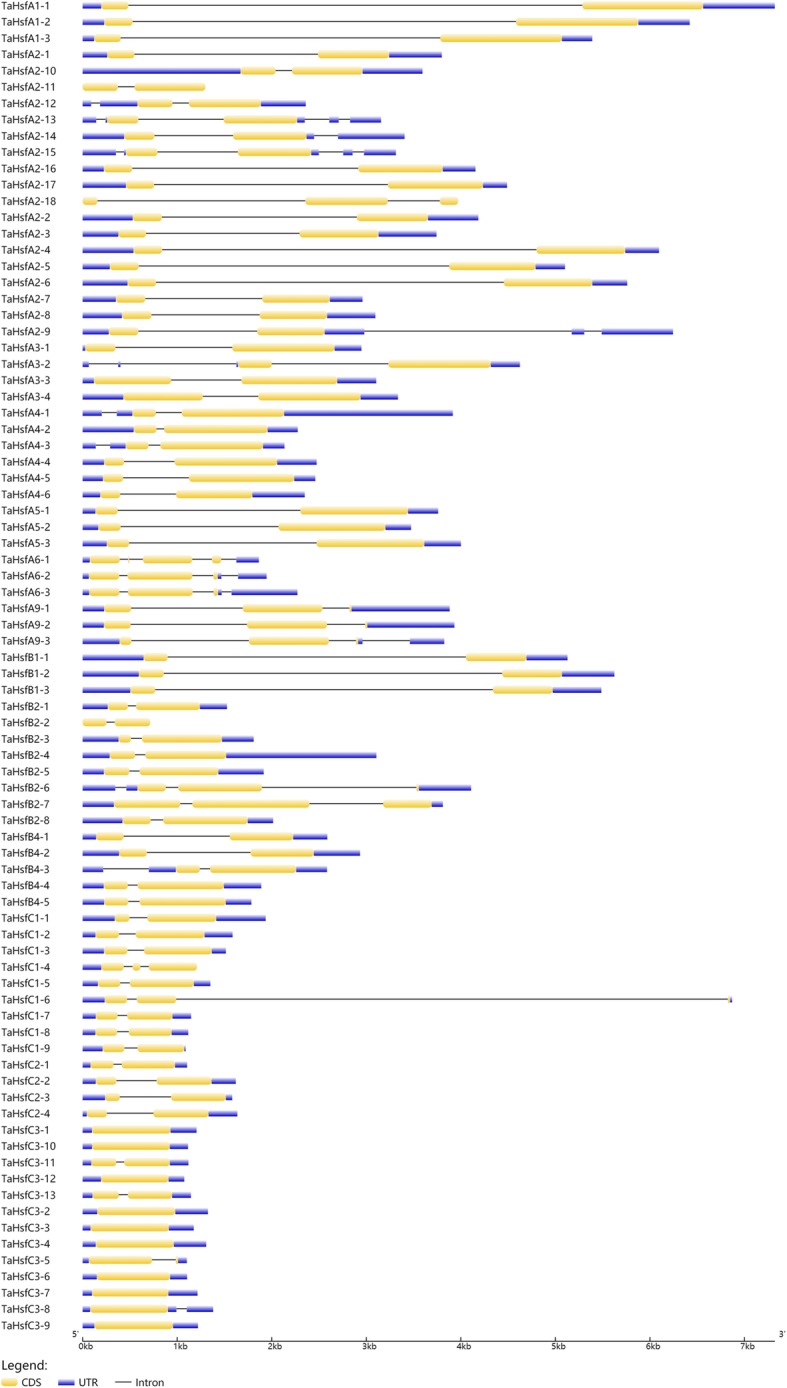
Intron-exon structures of wheat Hsf genes. The intron-exon structures were examined using the GSDS online tool. The exons, introns and untranslated regions are indicated by yellow boxes, black lines and blue boxes, respectively

To explore the structural diversity of the TaHsf members, the intron-exon organization of each TaHsf gene was analyzed by comparing the cDNA sequences with the corresponding genomic DNA sequence (Fig. 4, Additional file 4). Analysis of the intron-exon boundaries of all *TaHsfs* indicated a highly conserved organization, particularly within the homeologous TaHsf gene groups and same subclass TaHsf members. This observation further validated the precision of the classification. However, the number of exons and introns differed among the *TaHsfs*, 62 of the 82 Hsf genes exclusively contained two exons, whereas 10 Hsf genes contained three exons. We identified nine intronless genes, and 10 *TaHsfs* are consisted of a single exon, all of which belonged to subclass TaHsfC3. A previous study showed that most of Hsf DBDs underwent an insertion of a conserved intron, separating the DBD into two parts [9]. 53 of the 82 *TaHsfs* contained a single intron. Two TaHsfA2 members (*TaHsfA2–13*, *TaHsfA2–15*) contained four introns, which is the highest number in the TaHsf family genes. Additionally, the lengths of the TaHsf introns were highly variable, which ranged from 80 bp (*TaHsfA4–2*) to 5836 bp (*TaHsfC1–6*).

**Fig. 5:**
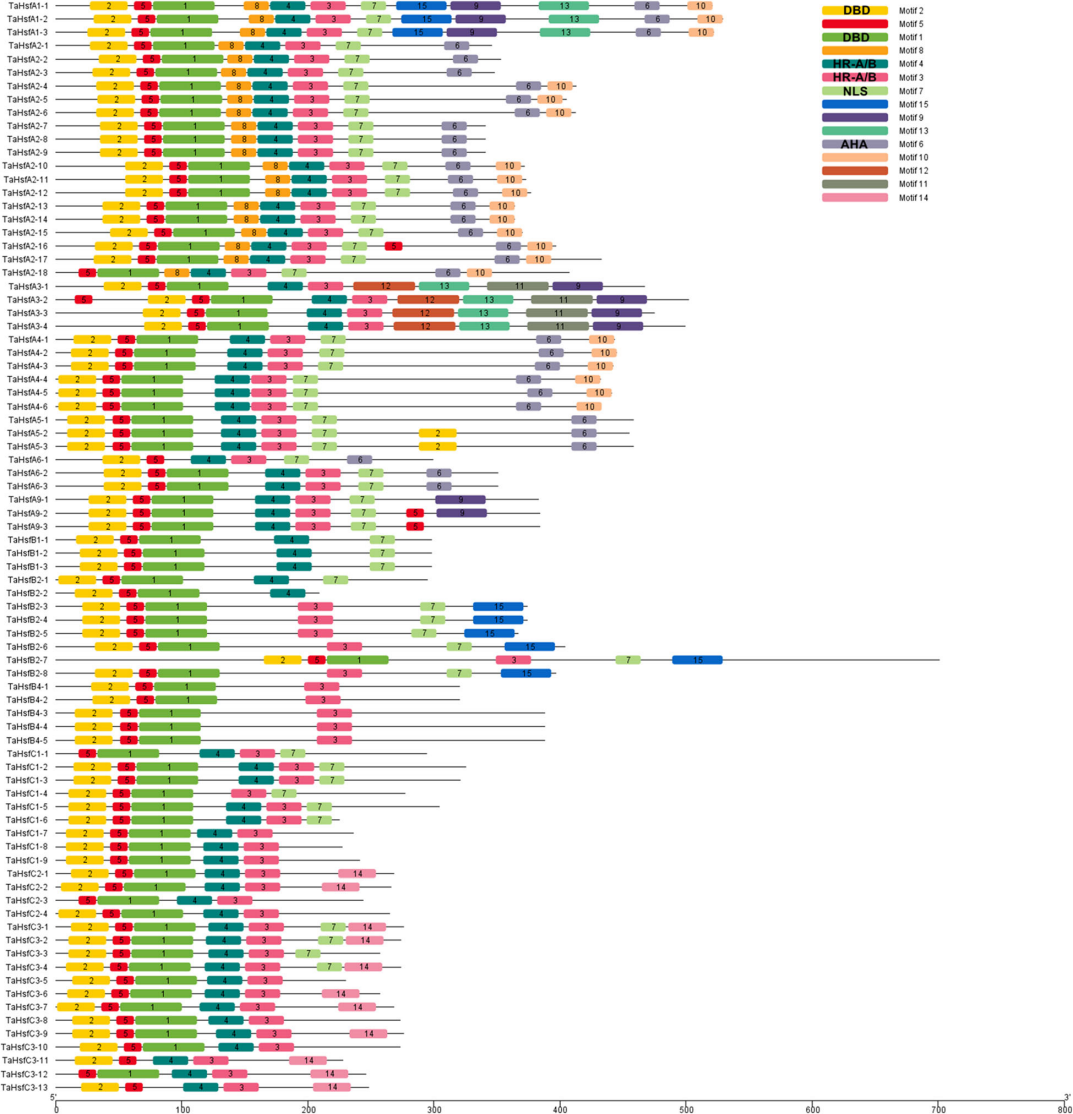
Motifs of the TaHsfs identified using MEME online tools. Fifteen motifs were identified (1–15) and shown using different colors; the same number and color in different Hsfs refer to the same motif. The names of the TaHsfs listed on the left side of the figure, and the motif sizes are indicated at the bottom of the figure

Conserved motif analysis was conducted using MEME, and 15 motifs were identified in TaHsf family members (Fig. 5, Additional file 5). To further determine the structural characteristics of the TaHsf family members, the SMART online tool was employed to predict the conserved domains. The DBD is the most conserved domain, which is composed of Motifs 1 and 2 in most TaHsfs (76 of 82). Six TaHsfs had shorter DBD domains. *TaHsfC1–1*, *TaHsfC2–3* and *TaHsfC3–12* only contained Motif 1; *TaHsfA6–1*, *TaHsfC3–11* and *TaHsfC3–13* only contained Motif 2. *TaHsfA2–18*, *TaHsfC1–1* and *TaHsfC2–3* had partial α1-helices; *TaHsfA6–1* and *TaHsfC3–11* did not contain β4-sheets; *TaHsfC3–12* and *TaHsfC3–13* lacked α1, β3 and β3, β4, respectively (Fig. 1A). The HR-A/B regions are indispensable domains that are characterized by the predicted coiled-coil structure, which had two typical motifs (3 and 4) in the TaHsf family. Motif 3 is the predominant motif, corresponding to the HR-A/B regions in 76 members of the TaHsf family. The DBD and HR-A/B domains were detected in all members of the TaHsf family. Additionally, Motif 7 corresponded to the NLS and Motif 6 represented the AHA. Overall, the structure of the TaHsf proteins was conserved among the TaHsf family members.

Depending on the balance between nuclear import and export, the intracellular distribution of Hsfs dynamically changes between the nucleus and cytoplasm [35]. The NES, NLS and ER membrane retention signals at the C-terminal of various Hsfs are required for regulating Hsfs subcellular localization. The NESs in 51 TaHsfs were predicted using NetNES, which included 25 TaHsfAs, 10 TaHsfBs and 16 TaHsfCs. The NLS domain was found in 50 members of TaHsf family, including 32 TaHsfAs, 11 TaHsfBs and 7 TaHsfCs; six and one bipartite NLS were found in classes B and C, respectively. Moreover, ER membrane retention signals were detected in 20 members of the TaHsf family. Additionally, the AHA domains, which were identified by sequence comparison, were detected in all TaHsfAs in the center of the C-terminal activation domains. However, these domains were not identified in TaHsfBs and TaHsfCs (Table 1). The tetrapeptide-LFGV domain, which is characteristic of HsfBs, was present in all TaHsfBs, except for TaHsfB2–2, in the C-terminal of the protein, and is predicted as the repressor motif of transcription [18].

### Transcription profiles of the Hsf genes in *T. aestivum*

**Fig. 6:**
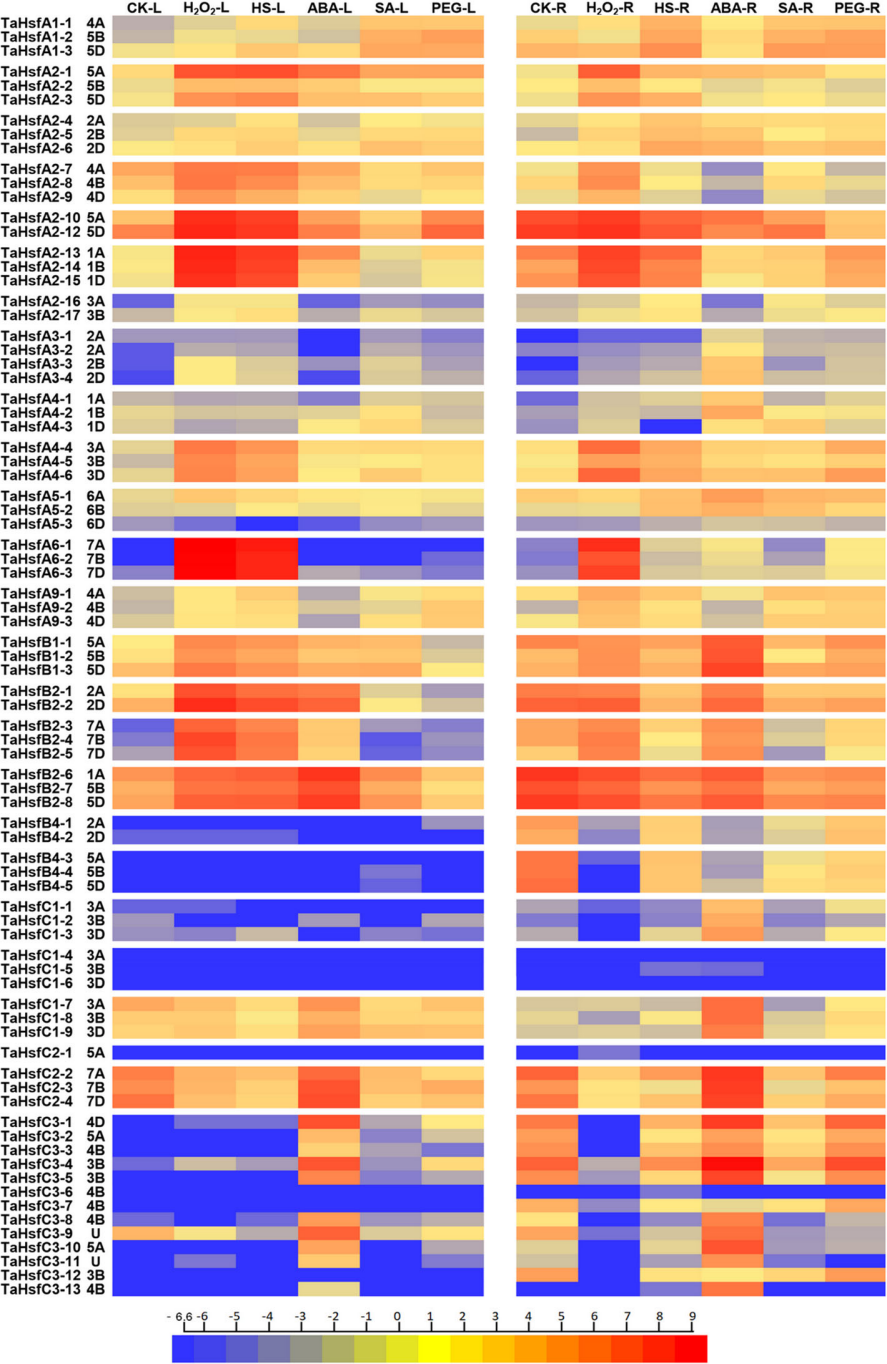
The transcription profiles of the TaHsf family genes in leaf (L) and root (R) tissues after H_2_O_2_, heat stress (HS), ABA, salicylic acid (SA) and polyethylene glycol (PEG) treatments. A heat map is drawn to illustrate the relative expression profiles of TaHsfs. Different colors correspond to log2 transformed values. Red or blue indicates higher or lower relative abundance of each transcript in each sample, respectively

Different abiotic stresses and phytohormone signal networks interact and share some common elements that form potential “nodes” for crosstalk [8]. The multifunctional plant Hsf genes are considered as nodes or cross-points that connect several pathways and simultaneously participate in various abiotic and phytohormone signaling pathways. Therefore, the results of transcriptome sequencing analysis under five different treatments (H_2_O_2_, heat stress, abscisic acid, salicylic acid, polyethylene glycol) and control in leaf and root tissues are shown in Fig. 6. Transcripts per million (TPM) values were used to measure the transcription level of the *TaHsfs* (Additional file 6). The transcription patterns of the 80 TaHsf genes revealed that *TaHsfs* are responsive to all the phytohormone and stress treatments to different degrees, and almost all TaHsf genes are expressed in the two tissues under different treatments, except for *TaHsfA3–1*, *TaHsfC1-(4–6)*, *TaHsfC2–1* and *TaHsfC3–6*. The transcription patterns of the TaHsf genes under different treatments and in different tissues significantly differed. However, *TaHsfs* from the same class, subclass, and most homoeologous TaHsf genes exhibited some degree of similarity in expression patterns.

In *T. aestivum* leaf tissues, the expression of TaHsf family was hardly detectable under normal conditions. *TaHsfAs* were the most inducible TaHsf genes that were strongly upregulated by both H_2_O_2_ and HS treatments. TaHsfC members were mainly induced by ABA treatment. TaHsfB members were induced by H_2_O_2_, HS and ABA treatments. The highest expression level in the TaHsf family were deteted in *TaHsfA6s* under H_2_O_2_ and heat treatment in leaf tissues, whereas the expression of *TaHsfA6s* could hardly be detected in the control and other treatments. Additionally, seven members of subclass A2 (*TaHsfA2–1*, *TaHsfA2–10*, *TaHsfA2–11*, *TaHsfA2–12*, *TaHsfA2–13*, *TaHsfA2–14* and *TaHsfA2–15*) were also strongly induced by H_2_O_2_ and heat treatments. The members of TaHsfB were responsive to H_2_O_2_, HS and ABA treatments. Subclass TaHsfB1 and *TaHsfB2-(1–5)* were more sensitive to H_2_O_2_ and heat treatments than ABA treatment. However, *TaHsfB2-(6–8)* were more sensitive to ABA treatment. The members of the TaHsfC subclass were only sensitive to ABA treatment. The members in subclass TaHsfA1 were more sensitive to SA and PEG (polyethylene glycol) treatments than the other subclasses. We could detect very weak expression of *TaHsfA3s* and *TaHsfA4-(1–3)*, but could hardly detect any expression of *TaHsfB4s* and *TaHsfC1-(1–6)* in the leaf tissues under all treatments and the control. Most of the TaHsf members were upregulated after treatment, except for *TaHsfC1–7*, *TaHsfC2–2*, *TaHsfC2–3* and *TaHsfC2–4*, which were downregulated after H_2_O_2_, HS, SA and PEG treatments.

In *T. aestivum* root tissues, the class C TaHsf family genes were more sensitive to ABA treatment, among them, *TaHsfC3–4* showed the highest expression levels. Both upregulated and downregulated response modes were observed in the *TaHsfBs* under ABA treatment, *TaHsfB1s* were up-regulated, whereas *TaHsfB2-(6–8)* were downregulated. Most of *TaHsfAs* were insensitive to ABA treatment, except *TaHsfA3s* and *TaHsfA4–1, TaHsfA4–2* which were only induced by ABA treatment in the root tissues. Members of TaHsfA were only sensitive to H_2_O_2_, which were upregulated after treatment. *TaHsfA2–10*, *TaHsfA2–12*, *TaHsfA2–13*, *TaHsfA2–14* and *TaHsfA2–15* exhibited relatively higher basic transcription levels in the root tissues under control conditions, and were only upregulated under H_2_O_2_ treatment, but were suppressed under the other treatments. SA treatment could only upregulate subclass TaHsfA1, as well as inhibit the expression of *TaHsfs*, which showed high basic transcription level. Most of the upregulation and high transcription level under PEG treatment were detected in *TaHsfC3s*, whereas the inhibited expression under PEG treatment was observed in *TaHsfC2–2*, *TaHsfC2–3* and *TaHsfC2–4*. The TaHsfA6 subclass exhibited H_2_O_2_ treatment-specific expression and was hardly detected in the other treatments and control. *TaHsfC1-(1–3)*, *TaHsfC1-(7–9)*, *TaHsfC3–10* and *TaHsfC3–13* exhibited ABA treatment-specific expression in the root tissues, among them, *TaHsfC1-(1–3)*, *TaHsfC3–7*, *TaHsfC3–12* and *TaHsfC3–13* exhibited root-specific expression and were hardly detected in the leaves. In general, *TaHsfs* in the root tissues had a higher expression diversity than the leaves.

### The expression pattern confirmation of selected TaHsf by qRT-PCR

**Fig. 7:**
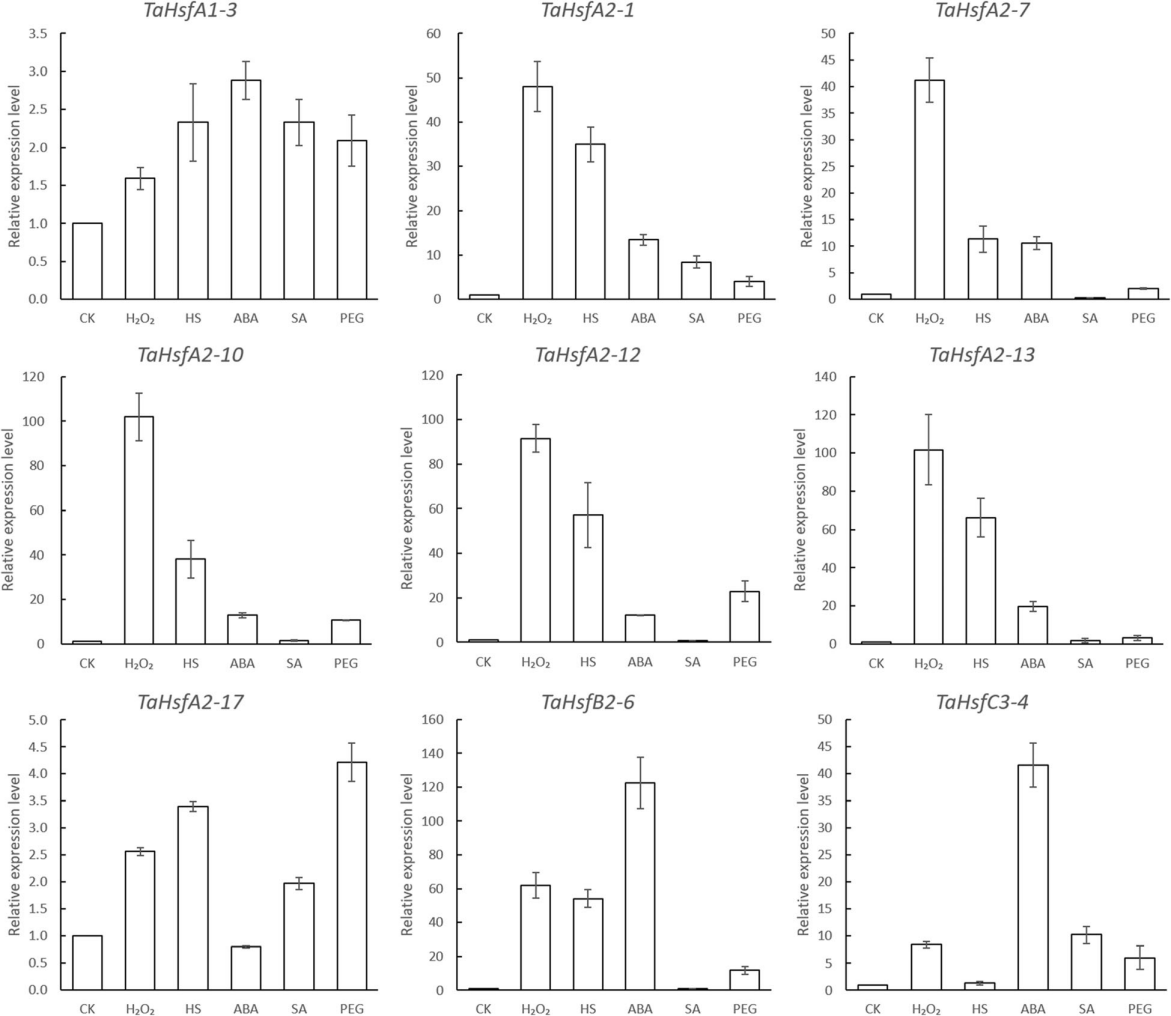
Relative expression level of selected TaHsfs analyzed by qRT-PCR under H_2_O_2_, heat stress, ABA, SA, PEG treatments. Each bar value represents the Mean ± SD of triplicate experiments

Nine TaHsf genes (*TaHsfA1–3, TaHsfA2–1, TaHsfA2–7, TaHsfA2–10, TaHsfA2–12, TaHsfA2–13, TaHsfA2–17, TaHsfB2–6, TaHsfC3–4*) from three major classes, were selected for examination of their expression pattern under control and treatments using qRT-PCR, which carried out using wheat seedling leaf treated with H_2_O_2_, HS, ABA, PEG, SA and control. The qRT-PCR result of selected *TaHsfs* expression pattern (Fig. 7) showed a high level of consistency with the results of RNA-seq analysis.

Both RNA-seq and qRT-PCR results showed that the *TaHsfA1–3* and *TaHsfA2–17* were insensitive to all treatments. The other TaHsfA members (*TaHsfA2–1, TaHsfA2–7, TaHsfA2–10, TaHsfA2–12, TaHsfA2–13*) could only be significantly induced by H_2_O_2_ and HS treatments, which could also be observed in RNA-seq analysis. The qRT-PCR results of *TaHsfB2–6* revealed that it could be upregulated under H_2_O_2_, HS and ABA treatments and insensitive to SA and PEG, and it is more sensitive to ABA than to H_2_O_2_ and HS treatments, both of which were consist with RNA-seq results. qRT-PCR and RNA-seq results showed that *TaHsfC3–4* could only be intensely induced by ABA treatment.

## Discussion

### Wheat has a large number of Hsf family genes

The common bread wheat, *T. aestivum*, has one of the most complex genomes known to science. The allohexaploid bread wheat genome consists of three closely related subgenomes (A, B and D) and an overall size of at least 17 billion bases [28]. Based on genetic similarity, the 21 pairs of wheat homeologous chromosomes are divided into seven homeologous groups, each containing one pair of chromosomes from the A, B and D subgenomes. The homeologous chromosome groups have similar sets of genes (syntenic genes) and homologous DNA sequences; therefore, the *T. aestivum* genome contains several groups of homeologous genes that have three highly identical sequences in each subgenome [36]. The huge *T. aestivum* genome and its seven homeologous chromosome groups provide a resource for generating a large TaHsf family. There are at least 56 *TaHsfs* in the *T. aestivum* genome. Because of the unavailability of the *T. aestivum* genome sequence at that time, the 56 TaHsfs were identified using a BLAST search of a limited database, which was collected from individual EST databases. Thus, the 56 TaHsfs that are identified and show high sequence redundancy, and some sequences were of partial length [37]. The present study employed an advanced method in identifying TaHsfs at the genome-wide level. A new HMM model of plant Hsf protein sequences was constructed, and 80 nonredundant, full-length *TaHsfs* were identified.

Studies on plant Hsf families have reported more than 20 species to date. The number of Hsf family genes widely differs among plants. There are 21 Hsfs-encoding genes in Arabidopsis, 24 in tomato [11], 25 in pepper [38], 25 in maize [27], 29 in Chinese white pear [39], 64 in *Brassica napus* [40]. Scharf et al. earlier suggested that the expansion of Hsfs in angiosperms is presumably the result of gene duplications and whole-genome duplications (WGDs) at different time points during evolution. Lineage-specific WGDs within the angiosperms are presumably the cause of the observed variations in the number of *Hsfs* among plant species [11]. The allohexaploid bread wheat genome was generated by the fusion of the *T. urartu* (subgenome A), *Aegilops speltoides* (subgenome B) and *A. tauschii* (subgenome D) genomes several hundred thousand years ago [41]. Approximately 60.1–61.3% of genes in the A, B and D subgenomes have orthologs in all the related diploid genomes [42]. Due to two rounds of allopolyploidy, the *TaHsfs* have tripled. Although the Hsf genes were always under strong purifying selection pressure [43], the allopolyploid process recently occurred, thus most of the *TaHsfs* were not eliminated by evolution. The present study found that 21 TaHsf homeologous gene groups consisted of 63 *TaHsfs*, which included three copies of genes that are located on each homeologous chromosome (A, B and D) and show high nucleotide sequence identity. These results indicate that the high number of *TaHsfs* in allohexaploid bread wheat was probably generated by allopolyploidy, whereas only 19 of the 82 TaHsf genes did not consist of an integrated homeologous group in the wheat genome, which was probably due to the incomplete genome sequence or gene loss during fractionation from ploidy [44]. The allotetraploid peanut [45] and cotton [46] also have relatively large numbers of Hsf genes in their genome. The number of Hsf genes in *Brassica napus* (genome AACC)*,* which was formed by recent interspecific hybridization, like wheat, has increased to 64 [40]. Therefore, the allopolyploid process, which resulted from the fusion of genomes, apparently contributed to the expansion of the plant Hsf family, which recently underwent allopolyploidy. In addition, allopolyploidy is proved to increase abiotic stress tolerance in plants [47, 48], and the larger number of members of the Hsf family may contribute to the higher abiotic stress tolerance.

### Classification and phylogenetic analysis of the TaHsf proteins

The present study showed that all 82 TaHsfs contained the highly conserved domains DBD and HR-A/B, which are essential for its transcriptional functions. All TaHsf genes could be divided into classes A, B and C based on the number of amino acid residues inserted between HR-A and HR-B.

For further classify TaHsfs into subclasses, an unrooted Neighbor-joining (NJ) tree was constructed using previously characterized Hsf and TaHsf families (Fig. 3). The TaHsfs were thus named based on the corresponding subclass name of the orthologous gene in model plants. Additionally, gene orthology analysis was also used as a preliminary method to investigate the function of TaHsfs [49]. The result showed that the class A were the predominant class. Whose number of genes and subclasses were the largest in the Hsf family genes in both monocots and dicots. Distinct differences in subclass species were also observed between monocots and dicots. Monocots tend to have a more complex class C species, whereas dicots have more complex class A and B, and dicots had more complex Hsf subclass species than monocots. The AtHsf family exclusively consisted of AtHsfA7s (AT3G51910, AT3G63350), AtHsfA8 (AT1G67970) and AtHsfB3 (AT2G41690), whereas monocots only included HsfC2. These findings were discordant to the results of Scharf et al., which was caused by the use of a different reference for Hsf subclass nomenclature [11]. The name and sequence of AtHsfs, OsHsfs and ZmHsfs were downloaded from PlantTFBD which do not include subclasses A7 and A8 in monocots. Therefore, we acquired the present results of Hsfs classification, which did not possess subclasses A7 and A8 in monocots. Our results are consistent with those of the classification and analysis of heat shock transcription factor family in maize which Hsfs classification were based on the data from PlantTFBD too [27].

The present study compared the Hsf family composition of three monocots, and all of them consisted of the same subclass species and proportion distribution, except for the novel subclass, TaHsfC3. Additionally, all of the same subclass genes from monocots were closely clustered. These results indicated that the subclass species and proportion distribution were relatively conserved in monocots, and each Hsf subclass may be indispensable for plants. We also found that the subclass HsfA2, HsfA6, HsfA9, HsfB1, HsfC1 genes from monocots and dicots were grouped into different clades, but the other subclass Hsf genes belonged to the same clades, which indicated that these same subclass Hsf genes from dicots and monocots were not closely related and thus may exist with relatively greater functional differences. A new clade with 13 TaHsfC members, hereby named TaHsfC3, did not have any orthologous genes in the model plant Hsf family. These new subclass genes might have different functions or roles in the stress signaling pathway in wheat.

### Conserved structure analysis of TaHsfs

Similar to the Hsf families in other plants, the structure of the TaHsf proteins are well conserved. The DBD domain of plant Hsfs, which is characterized as a key domain, is encoded by two regions that are divided by an evolutionarily conserved intron, which was inserted immediately adjacent to the HTH DNA-binding motif [11]. This intron was detected in most DBD domains of TaHsfs, whereas no intron was found in *TaHsfC3–1*, *TaHsfC3–2*, *TaHsfC3–3*, *TaHsfC3–4*, *TaHsfC3–6*, *TaHsfC3–7*, *TaHsfC3–8*, *TaHsfC3–9*, *TaHsfC3–10* and *TaHsfC3–12* from the novel subclass TaHsfC3, as shown by their gene structure (Fig. 4). Previous study also shown that *BnaHsf64* and *BnaHsf64* without any introns inserted into the DBD domain [40]. Sequence alignment and MEME analysis identified six TaHsfs with an incomplete DBD domain (Figs. 1 and 5), whereas these genes were proven to be responsive to stress treatments (Fig. 6). These findings indicated that these TaHsfs with incomplete DBD domains can participate in plant stress responses.

### Diverse transcription patterns of TaHsf family genes under different treatments

Plant Hsf genes not only could respond to abiotic stresses, but also to phytohormones. Genome-wide expression profiling of plant Hsf families under various abiotic stresses and phytohormones has been extensively studied in different tissues [43, 50, 51]. It was essential to investigate the expression profile of the TaHsf family under different stresses and phytohormones, before further studying a specific TaHsf gene. Our work revealed that the TaHsf family genes could respond to three stresses and two phytohormones, which suggests that TaHsfs might not only improve the heat tolerance of plants, but could also play a crucial role in increasing tolerance to various abiotic stresses as well as in enhancing signaling pathways. Organ-specific expression of *TaHsfs* was also observed between leaves and roots. Most of the TaHsf homoeolgous groups had a similar expression pattern or mode under different treatments, for they shared a high level of sequence identities. The similar expression pattern was also found in the research on *TaHsfC2a* homoeolgous group [52]. But the diverse expression level or pattern also been observed, such as *TaHsfA2–1* is more sensitive to H_2_O_2_ than *TaHsfA2–2* and *TaHsfA2–3* in both leaf and root tissue; *TaHsfA5–1*, *TaHsfA5–2* could be responsive to treatments in both leaf and root tissue, but *TaHsfA5–3* showed no detectable expression under control and treatments. The duplicated Hsf genes were under strong purifying selection pressure [43], and the wheat genome often undergoes extensive genomic rearrangement, which may cause TaHsf functional differentiation or transcriptional silencing [53, 54]. The diverse expression pattern of homoeologous genes are also suggested to facilitate abiotic acclimation of wheat [55].

The *TaHsfs* in leaf tissues showed hypersensitivity to H_2_O_2_ treatment, particularly in terms of *TaHsfAs* and *TaHsfB1s*, which exhibited the most significantly upregulated expression. It has been postulated that plant Hsfs act as sensors of ROS levels, resulting in Hsf activation and subsequent expression of other regulatory genes, including other *Hsfs* [56, 57]. H_2_O_2_ activates the Ca^2+^ signaling pathway at the cell surface, as well as entering the cell through PIP water channels and then activating Ca^2+^ signaling intracellularly [58]. Ca^2+^ /calmodulin (CaM) could directly regulate the activation of *Hsfs* by phosphorylation of *Hsfs* [59]. This indicates that H_2_O_2_ may be the direct upstream regulatory component of *Hsfs*. *TaHsfs* were most insensitive to SA and PEG treatments in the leaf tissues. *TaHsfA1s* were slightly upregulated, whereas the expression of other *TaHsfs* could be hardly detected under SA treatment. SA has been found to be involved in both basal and acquired thermotolerance in plants and activates various plant defense responses [60]. Similar results have been observed in tomato, *HsfA1* could be induced by SA treatment, but not *HsfA2*. However, SA treatment could induce the expression of Hsp genes and increase the heat tolerance of plants by enhancing Hsf DNA-binding ability [12]. *TaHsfs* in leaf tissues were insensitive to PEG treatment, whereas these were relatively sensitive in the root tissues. These results have also been observed in the soybean Hsf family under PEG treatment [51]. This is consistent with the fact that the leaves are the first to experience heat stress, whereas the roots are the first organs that perceive drought stress. In this study, 6 h of PEG treatment was not enough to lead to active drought stress responses in the leaf tissues. The expression profiles of *TaHsfs* revealed a specific responsive pattern to different treatments at the class level. Especially in the leaf tissues, *TaHsfAs* exhibited sensitivity to H_2_O_2_ and HS treatment, and *TaHsfCs* were sensitive to ABA treatment, whereas *TaHsfBs* were sensitive to H_2_O_2_, HS and ABA treatments. These results suggested the TaHsfs were divided into different classes, which may have specific functions of stress resistance. It also could provide a reference for discovering the specific function of each TaHsf gene. Compared to the other three Hsf family members, the rice Hsf family is most closely related to the TaHsf family, which was also observed in other gene family studies [61, 62]. Therefore, we compared the expression patterns of orthologous Hsf genes between wheat and rice in response to different treatments, which revealed that both of these are most sensitive to HS and H_2_O_2_, as indicated by the upregulation of Hsfs in the seedling leaves. TaHsfs were more sensitive to H_2_O_2_, whereas OsHsfs were more sensitive to HS treatment. In terms of specific Hsf genes, *OsHsfA2a* (Os03g0745000), *OsHsfA6a* (Os06g0565200) and *OsHsfB2a* (Os04g0568700) were the most inducible upregulated genes in rice, *TaHsfA2–10*, *TaHsfA2–12* as orthologs of *OsHsfA2a* and *TaHsfA6–1*, *TaHsfA6–2*, *TaHsfA6–3* as orthologs of *OsHsfA6a* and *TaHsfB2–1, TaHsfB2–2*, *TaHsfB2–3* as orthologs of *OsHsfB2a* were also significantly regulated with HS and H_2_O_2_ treatment. These results suggested that there were certain similarities in the stress response patterns between these two plants, and these Hsf genes may play an essential role in abiotic tolerance in plants. We also observed different expression patterns between rice and wheat orthologous Hsf genes such as *TaHsfA2–13*, *TaHsfA2–14* and *TaHsfA2–15*, which were significantly upregulated under HS and H_2_O_2_ treatments, whereas the ortholog *OsHsfA2c* (Os10g28340) was insensitive to these treatments [50].

ABA is known to play important roles in regulating plant responses to various abiotic stresses, particularly those involving dehydration such as drought, salinity and cold stress [58]. ABA can improve tolerance to various abiotic stresses in plants through the regulation of *Hsfs* and *Hsps*. Our result revealed that the ABA-inducible *TaHsfs* mainly belong to classes B and C regardless of tissue. *TaHsfC2–2*, *TaHsfC2–3* and *TaHsfC2–4* were constitutively expressed in the leaves and roots and were only up-regulated by ABA treatment, whereas downregulated by other treatments. *TaHsfs* in subclass C3 were only induced by ABA treatment in the leaves. Subclasses TaHsfA3, *TaHsfA4–1* and *TaHsfA4–2* were only induced by ABA in the root tissues, but were hardly detected in the leaves. Huang et al. previously reported that *AtHsfA6a* (AT5G43840) and *AtHsfA6b* (AT3G22830) were induced by ABA, but not HS, and these genes are involved in the ABA signal pathway and ABA-mediated thermotolerance and drought tolerance [13]. These findings suggested that *TaHsfs* might also play a crucial role in the response and acclimation to drought stress in wheat, and ABA-induced *TaHsfs* seem to hold an independent responsive pathway that differs from heat and oxidative stress. The spatial expression of *Hsfs* has been investigated in several previous studies. *OsHSFA6b* (Os01g39020) and *OsHSFA9* (Os03g12370) showed seed-specific expression in rice [63]. *AtHsfA9* (AT5G54070) is exclusively expressed during the late stages of seed development [64]. *GmHsf-02* is uniquely expressed in soybean roots [51]. In this work, we found that the expression pattern of *TaHsfs* significantly differed between leaves and roots. Several *TaHsfs* (*TaHsfA2–10*, *TaHsfA2–12*, *TaHsfB2–6*, *TaHsfB2–7*, *TaHsfB2–8*, *TaHsfC2–2* and *TaHsfC3–4*) exhibit high basic expression levels in the roots, which in turn could hardly be detected in the leaves. Subclass TaHsfA6 was the most inducible TaHsf genes, which were upregulated by both H_2_O_2_ and heat treatment in the leaves, whereas it was only responsive to H_2_O_2_ treatment in the roots. These findings suggest that *TaHsfA6s* may function specifically in the oxidative stress signaling pathway, but not to osmotic stress. Subclass TaHsfB4 had a high basic expression level in root tissues, which dramatically decreased with all treatments, indicating that *TaHsfB4s* may be involved in root development.

## Conclusions

A new Hsf protein HMM model constructed by the Hsf protein sequence of model monocot (*O. sativa*) and dicot (*A. thaliana*) plants was applied to identify novel Hsfs in the proteome of *T. aestivum*. A total of 82 non-redundant, full-length *TaHsfs* were identified and localized. Structural characteristics and phylogenetic analysis of *T. aestivum, O. sativa*, *A. thaliana* and *Z. mays* were performed to classify TaHsf genes into three major classes and further into 13 subclasses. A novel subclass TaHsfC3 was identified in this study. The allopolyploid process, which occurred after the fusion of genomes, may have contributed to the expansion of the TaHsf genes. Furthermore, the *TaHsfs* expression profiles by RNA-seq revealed that *TaHsfs* are not only responsive to abiotic stresses but also to specific phytohormones. Additionally, these *TaHsfs* exhibited class-, subclass- and organ-specific responsive patterns. The new multifunctional *TaHsfs* characterized in this study improves our understanding of the response and acclimation of plants to multifactorial and combinational abiotic stresses, as well as provides candidate gene resources for further investigations on abiotic stress tolerance in crops.

## Methods

### Data collection and identification of Hsf genes

The *T. aestivum* (bread wheat) genome sequence data of TGACv1 were obtained from the plant genome database [31]. The Hsf amino acid sequences and names of *A. thaliana*, *O. sativa* and *Z. mays* were downloaded from PlantTFBD [65]. The HMM model of Hsf was constructed using hmmer3.0 based on the Hsf amino acid sequences of *A. thaliana* and *O. sativa* [66]*.* The HMM model of Hsf was used as query to search all possible Hsf protein sequences in the wheat proteome database using BLASTP (E < 0.001). The integrated DBD and HR-A/B domains in the putative wheat Hsfs were examined using SMART [67]. Candidate proteins without the HR-A/B or DBD domains were excluded from further analysis. The NLS and NES domains in the wheat Hsfs were predicted using cNLS Mapper and NetNES 1.1 [68, 69]. The prediction of AHA domains was based on the conserved AHA motif sequence (FWxxF/L, F/I/L) [70]. Protein isoelectric point (pI) and molecular weight (Mw) were calculated using ExPasy [71].

A total of 79 *TaHsfs* were mapped onto the 21 wheat chromosomes according to the information in the wheat database using MapGene2Chromosom [72]. Homeologous gene groups were identified by three high-identity nucleotide sequence [36].

### Multiple sequence alignment and phylogenetic analysis

The phylogenetic tree was constructed using the neighbor joining (NJ) method in MEGA (version 5.0) [73]. NJ analysis was conducted with the pairwise deletion option and the Possion correction. For statistical reliability, bootstrap analysis was performed with 1000 replicates to assess statistical support for each node. For increasing readability of the phylogenetic tree, the N-terminal parts of the proteins containing the DBD, the HR-A/B and the linker between these two regions of TaHsfs were used in the analysis.

### Orthologous gene identification and structure analysis

Orthologous gene pairs were identified based on (1) the best hit between *A. thaliana*, *O. sativa* and *T. aestivum*, (2) the position in the phylogenetic tree (bootstrap value > 50), and (3) the identity between orthologous gene pairs (> 90%). Intron-exon organization of the Hsf genes in wheat is illustrated using Gene Structure Display Server program [74] by alignment of the cDNAs with their corresponding genomic DNA sequences. The MEME program [75] was used for identification of conserved motifs, with the following parameters: the optimum motif widths: 6–50 amino acid residues and any number of repetitions: maximum number of motifs:15.

### Plant materials and treatments

Cang 6005, the thermo-insensitive cultivated wheat provided by CangZhou Academy of Agriculture and Forestry Sciences, was used in this study. The selected seeds were surface-sterilized and repeatedly rinsed with tap water, then seeded in Hoagland nutrient solution after immersion and imbibition for 12 h, and cultivated in the incubator at 25 °C with a 16 h/8 h photoperiod/dark period. Six two-week-old homogeneous seedlings groups, each of which included 50 seedlings from five biological replicates, were subjected to different treatments, including 10 mM H_2_O_2_ for 90 min, heat (37 °C) for 60 min, 0.2 mM ABA for 12 h, 20% PEG6000 for 6 h, 0.8 mM SA for 1.5 h and control. The second leaf was sampled for the experiment. Pooled samples from each group were collected and immediately frozen in liquid nitrogen for RNA extraction.

### RNA isolation and RNA-Seq analysis

Total RNA of each treatment was isolated from 50 seedlings from five biological replicates using the Total RNA Extractor (TRIzol) kit (B511311, Sangon, China), and RNase-free DNase I was used to remove genomic DNA contamination. RNA integrity was estimated with an Agilent 2100 Bioanalyzer (Agilent Technologies, CA, USA). Approximately 2 μg of RNA from each sample was used as input material for RNA sample preparation. VAHTSTM mRNA-seq V2 Library Prep Kit for Illumina® was used to prepare the sequencing libraries. The HiSeq XTen sequencers (Illumina, San Diego, CA, USA) were used in paired-end sequencing of the library. FastQC (version 0.11.2) was applied to evaluate the quality of sequenced data. Subsequently, the raw reads were screened using Trimmomatic (version 0.36). HISAT2 (version 2.0) was used to map the clean reads to the reference genome using default parameters. StringTie (version 1.3.3b) was used to calculate the gene expression abundance of the transcripts. TPM was used as transcript measurement, which eliminates the effect of gene sequencing discrepancies and the lengths enabled direct comparisons of gene expression among samples. Differentially expressed genes (DEGs) were identified by DESeq2 (version 1.12.4).

### RNA isolation, cDNA synthesis & qRT-PCR analysis

To confirm the expression of representative of TaHsf genes, total RNA was isolated using Redzol (Beijing SBS Genetech Co.,Ltd.). The residual DNA was removed by DNase I (TaKaRa). For reverse transcription, the first-strand cDNA was synthesized using a PrimeScript™ first-strand cDNA synthesis kit (TaKaRa). Quantitative real-time PCR (qRT-PCR) for examination of the *TaHsfs* were performed with the SYBR Premix ExTaqTM kit (TaKaRa) and an ABI 7500 (Applied Biosystem). Gene-specific and internal reference gene TaRP15 primers were listed in Additional file 7. The qRT-PCR program was set as the following: predenaturation at 95 °C for 30 s; denaturation at 95 °C for 5 s; annealing/extension at 60 °C for 34 s, 40 cycles. 2^−ΔΔCt^ method was used to analyze the data. The expression level of *TaHsfs* in leaf was set as 1. Three biological replicates were included for each group of experiments, and three technical replicates were included for each biological sample. The data were represented by mean value ± standard error of three biological replicates, as the previous described [76].

## Additional files

Additional file 1: **Table S1.** Sequences and transcript IDs of novel TaHsfs. (XLSX 73 kb)

Additional file 2: **Table S2.** Physical location of TaHsfs. (XLSX 12 kb)

Additional file 3: **Table S3.** Orthologous gene pairs of Hsf family of *Triticum aestivum, Oryza sativa, Zea mays* and *Arabidopsis thaliana*. (XLS 25 kb)

Additional file 4: **Table S4.** Exon-intron structures of TaHsfs. (XLSX 18 kb)

Additional file 5: **Table S5.** Motif sequences identified by MEME tools. (DOC 838 kb)

Additional file 6: **Table S6.** Heat map of the expression of TaHsfs in the leaves and roots using five different treatments. (XLSX 16 kb)

Additional file 7: **Table S7.** Gene-specific and internal reference gene *TaRP15* primers used for qRT-PCR. (XLSX 10 kb)

## Abbreviations

ABA: Abscisic Acid
AHA: Activator motif
DBD: DNA Binding Domain
HMM: Hidden Markov Model
HR: Heptad Repeats
HS: Heat Shock
Hsf: Heat shock factor
Hsp: Heat shock protein
HTH: helix-turn-helix
NES: Nuclear Export Signal
NJ: Neighbor-joining
NLS: Nuclear Localization Signal
OD: Oligomerization Domain
PEG: Polyethylene Glycol
qRT-PCR: quantitative Real-Time PCR
ROS: Reactive Oxygen Species
SA: Salicylic Acid
TPM: Transcripts Per Million

## References

[1] Wardlaw I, Dawson I, Munibi P, Fewster R (1989). The tolerance of wheat to high temperatures during reproductive growth. I. Survey procedures and general response patterns. Aust J Agric Res.

[2] Hansen J, Sato M, Ruedy R (2012). Perception of climate change. Proc Natl Acad Sci U S A.

[3] Stone P, Nicolas M (1995). Effect of timing of heat stress during grain filling on two wheat varieties differing in heat tolerance. I. Grain growth. Aust J Plant Physiol.

[4] Al-Whaibi MH (2011). Plant heat-shock proteins: a mini review. J King Saud Univ-Sci.

[5] Zhou R, Li B, Liu H, Sun D (2009). Progress in the participation of Ca^2+^–calmodulin in heat shock signal transduction. Prog Nat Sci.

[6] Nakashima K, Takasaki H, Mizoi J, Shinozaki K, Yamaguchi-Shinozaki K (2012). NAC transcription factors in plant abiotic stress responses. Biochim Biophys Acta.

[7] Lata C, Prasad M (2011). Role of DREBs in regulation of abiotic stress responses in plants. J Exp Bot.

[8] Akhtar M, Jaiswal A, Taj G, Jaiswal J, Qureshi M, Singh N (2012). DREB1/CBF transcription factors: their structure, function and role in abiotic stress tolerance in plants. J Genet.

[9] Nover L, Bharti K, Döring P, Mishra SK, Ganguli A, Scharf KD (2001). Arabidopsis and the heat stress transcription factor world: how many heat stress transcription factors do we need?. Cell Stress Chaperones.

[10] Mittler R (2002). Oxidative stress, antioxidants and stress tolerance. Trends Plant Sci.

[11] Scharf K-D, Berberich T, Ebersberger I, Nover L (2012). The plant heat stress transcription factor (Hsf) family: structure, function and evolution. Biochim Biophys Acta.

[12] Snyman M, Cronjé MJ (2008). Modulation of heat shock factors accompanies salicylic acid-mediated potentiation of Hsp70 in tomato seedlings. J Exp Bot.

[13] Huang Y, Niu C, Yang C, Jinn T (2016). The heat-stress factor HSFA6b connects ABA signaling and ABA-mediated heat responses. Plant Physiol.

[14] Scharf K-D, Rose S, Zott W, Schöffl F, Nover L, Schöff F (1990). Three tomato genes code for heat stress transcription factors with a region of remarkable homology to the DNA-binding domain of the yeast HSF. EMBO J.

[15] Baniwal SK, Bharti K, Chan KY, Fauth M, Ganguli A, Kotak S, Mishra SK, Nover L, Port M, Scharf K-D (2004). Heat stress response in plants: a complex game with chaperones and more than twenty heat stress transcription factors. J Biosci.

[16] Döring P, Treuter E, Kistner C, Lyck R, Chen A, Nover L (2000). The role of AHA motifs in the activator function of tomato heat stress transcription factors HsfA1 and HsfA2. Plant Cell.

[17] Ikeda M, Ohme-Takagi M (2009). A novel group of transcriptional repressors in Arabidopsis. Plant Cell Physiol.

[18] Czarneckaverner E, Pan S, Salem T, Gurley WB (2004). Plant class B HSFs inhibit transcription and exhibit affinity for TFIIB and TBP. Plant Mol Biol.

[19] Liu H, Charng Y (2012). Acquired thermotolerance independent of heat shock factor A1 (HsfA1), the master regulator of the heat stress response. Plant Signal Behav.

[20] Yoshida T, Ohama N, Nakajima J, Kidokoro S, Mizoi J, Nakashima K, Maruyama K, Kim J-M, Seki M, Todaka D (2011). Arabidopsis HsfA1 transcription factors function as the main positive regulators in heat shock-responsive gene expression. Mol Gen Genomics.

[21] Charng Y, Liu H, Liu N, Chi W, Wang C, Chang S, Wang T (2007). A heat-inducible transcription factor, HsfA2, is required for extension of acquired thermotolerance in Arabidopsis. Plant Physiol.

[22] Ogawa D, Yamaguchi K, Nishiuchi T (2007). High-level overexpression of the Arabidopsis HsfA2 gene confers not only increased themotolerance but also salt/osmotic stress tolerance and enhanced callus growth. J Exp Bot.

[23] Liu H, Charng Y (2013). Common and distinct functions of Arabidopsis class A1 and A2 heat shock factors in diverse abiotic stress responses and development. Plant Physiol.

[24] Baniwal SK, Chan KY, Scharf K-D, Nover L (2007). Role of heat stress transcription factor HsfA5 as specific repressor of HsfA4. J Biol Chem.

[25] Xiang J, Ran J, Zou J, Zhou X, Liu A, Zhang X, Peng Y, Tang N, Luo G, Chen X (2013). Heat shock factor OsHsfB2b negatively regulates drought and salt tolerance in rice. Plant Cell Rep.

[26] Schmidt R, Schippers JH, Welker A, Mieulet D, Guiderdoni E, Mueller-Roeber B (2012). Transcription factor OsHsfC1b regulates salt tolerance and development in *Oryza sativa ssp. japonica*. AoB Plants.

[27] Zhu SW, Tang XL, Chu ZX, Jiang HY, Lin YX, Cheng BJ (2011). Genome-wide identification, classification and analysis of heat shock transcription factor family in maize. BMC Genomics.

[28] Zimin AV, Puiu D, Hall R, Kingan S, Clavijo BJ, Salzberg SL (2017). The first near-complete assembly of the hexaploid bread wheat genome, *Triticum aestivum*. Gigascience.

[29] Huang S, Sirikhachornkit A, Su X, Faris J, Gill B, Haselkorn R, Gornicki P (2002). Genes encoding plastid acetyl-CoA carboxylase and 3-phosphoglycerate kinase of the Triticum/Aegilops complex and the evolutionary history of polyploid wheat. Proc Natl Acad Sci U S A.

[30] Salamini F, Ozkan H, Brandolini A, Schäferpregl R, Martin W (2002). Genetics and geography of wild cereal domestication in the near east. Nat Rev Genet.

[31] Clavijo BJ, Venturini L, Schudoma C, Accinelli GG, Kaithakottil G, Wright J, Borrill P, Kettleborough G, Heavens D, Chapman H (2017). An improved assembly and annotation of the allohexaploid wheat genome identifies complete families of agronomic genes and provides genomic evidence for chromosomal translocations. Genome Res.

[32] Kumar RR, Rai RD (2014). Can wheat beat the heat: understanding the mechanism of thermotolerance in wheat ( Triticum aestivum L.): a review. Cereal Res Commun.

[33] Asseng S, Foster I, Turner NC (2011). The impact of temperature variability on wheat yields. Glob Chang Biol.

[34] Ni Z, Li H, Zhao Y, Peng H, Hu Z, Xin M, Sun Q (2018). Genetic improvement of heat tolerance in wheat: recent progress in understanding the underlying molecular mechanisms. Crop J.

[35] Heerklotz D, Döring P, Bonzelius F, Winkelhaus S, Nover L (2001). The balance of nuclear import and export determines the intracellular distribution and function of tomato heat stress transcription factor HsfA2. Mol Cell Biol.

[36] Marcussen T, Sandve SR, Heier L, Spannagl M, Pfeifer M, Jakobsen KS, Wulff BB, Steuernagel B, Mayer KF, Olsen OA (2014). Ancient hybridizations among the ancestral genomes of bread wheat. Science.

[37] Xue GP, Sadat S, Drenth J, Mcintyre CL (2014). The heat shock factor family from Triticum aestivum in response to heat and other major abiotic stresses and their role in regulation of heat shock protein genes. J Exp Bot.

[38] Guo M, Lu J, Zhai Y, Chai W, Gong Z, Lu M (2015). Genome-wide analysis, expression profile of heat shock factor gene family (CaHsfs) and characterisation of CaHsfA2 in pepper (*Capsicum annuum* L.). BMC Plant Biol..

[39] Qiao X, Li M, Li L, Yin H, Wu J, Zhang S (2015). Genome-wide identification and comparative analysis of the heat shock transcription factor family in Chinese white pear (Pyrus bretschneideri) and five other Rosaceae species. BMC Plant Biol.

[40] Zhu X, Huang C, Zhang L, Liu H, Yu J, Hu Z, Hua W (2017). Systematic analysis of Hsf family genes in the Brassica napus genome reveals novel responses to heat, drought and high CO_2_ stresses. Front Plant Sci.

[41] Petersen G, Seberg O, Yde M, Berthelsen K (2006). Phylogenetic relationships of Triticum and Aegilops and evidence for the origin of the a, B, and D genomes of common wheat (Triticum aestivum). Molecular Phylogenetics & Evolution.

[42] Mayer KFX, Rogers J, Doležel J, Pozniak C, Eversole K, Feuillet C, Gill B, Friebe B, Lukaszewski AJ, Sourdille P (2014). A chromsome-based draft sequence of the hexaploid bread wheat (Triticum aestivum) genome. Science..

[43] Dossa K, Diouf D, Cissé N (2016). Genome-wide investigation of Hsf genes in sesame reveals their segmental duplication expansion and their active role in drought stress response. Front Plant Sci.

[44] Lynch M, Force A (2000). The probability of duplicate gene preservation by subfunctionalization. Genetics..

[45] Wang P, Song H, Li C, Li P, Li A, Guan H, Hou L, Wang X (2017). Genome-wide dissection of the heat shock transcription factor family genes in arachis. Front Plant Sci.

[46] Wang J, Sun N, Deng T, Zhang L, Zuo K (2014). Genome-wide cloning, identification, classification and functional analysis of cotton heat shock transcription factors in cotton (Gossypium hirsutum). BMC Genomics.

[47] Chao DY, Dilkes B, Luo H, Douglas A, Yakubova E, Lahner B, Salt DE (2013). Polyploids exhibit higher potassium uptake and salinity tolerance in Arabidopsis. Science..

[48] Yang C, Zhao L, Zhang H, Yang Z, Wang H, Wen S, Zhang C, Rustgi S, Von WD, Liu B (2014). Evolution of physiological responses to salt stress in hexaploid wheat. Proc Natl Acad Sci U S A.

[49] Wang M, Vannozzi A, Wang G, Liang Y-H, Tornielli GB, Zenoni S, Cavallini E, Pezzotti M, Cheng Z-MM (2014). Genome and transcriptome analysis of the grapevine (Vitis vinifera L.) WRKY gene family. Horticulture. Research..

[50] Mittal D, Chakrabarti S, Sarkar A, Singh A, Grover A (2009). Heat shock factor gene family in rice: genomic organization and transcript expression profiling in response to high temperature, low temperature and oxidative stresses. Plant Physiology & Biochemistry.

[51] Li P, Yu T, He G, Chen M, Zhou Y, Chai S, Xu Z, Ma Y (2014). Genome-wide analysis of the Hsf family in soybean and functional identification of GmHsf-34 involvement in drought and heat stresses. BMC Genomics.

[52] Hu XJ, Chen D, Lynne Mclntyre C, Fernanda Dreccer M, Zhang ZB, Drenth J, Kalaipandian S, Chang H, Xue GP (2018). Heat shock factor C2a serves as a proactive mechanism for heat protection in developing grains in wheat via an ABA-mediated regulatory pathway. Plant Cell Environ.

[53] Chen ZJ (2007). Genetic and epigenetic mechanisms for gene expression and phenotypic variation in plant polyploids. Annu Rev Plant Biol.

[54] Leach LJ, Belfield EJ, Jiang C, Brown C, Mithani A, Harberd NP (2014). Patterns of homoeologous gene expression shown by RNA sequencing in hexaploid bread wheat. BMC Genomics.

[55] Liu Z, Xin M, Qin J, Peng H, Ni Z, Yao Y, Sun Q (2015). Temporal transcriptome profiling reveals expression partitioning of homeologous genes contributing to heat and drought acclimation in wheat ( Triticum aestivum L.). BMC Plant Biol.

[56] Gadjev I, Vanderauwera S, Gechev TS, Laloi C, Minkov IN, Shulaev V, Apel K, Inzé D, Mittler R, Van BF (2006). Transcriptomic footprints disclose specificity of reactive oxygen species signaling in Arabidopsis. Plant Physiol.

[57] Miller G, Mittler R (2006). Could heat shock transcription factors function as hydrogen peroxide sensors in plants?. Ann Bot.

[58] Zhu JK (2016). Abiotic stress signaling and responses in plants. Cell..

[59] Liu HT, Gao F, Li GL, Han JL, Liu DL, Sun DY, Zhou RG (2010). The calmodulin-binding protein kinase 3 is part of heat-shock signal transduction in Arabidopsis thaliana. Plant J.

[60] Larkindale J, Knight MR (2002). Protection against heat stress-induced oxidative damage in Arabidopsis involves calcium, abscisic acid, ethylene, and salicylic acid. Plant Physiol.

[61] Guo J, Islam MA, Lin H, Ji C, Duan Y, Liu P, Zeng Q, Day B, Kang Z, Guo J (2018). Genome-wide identification of cyclic nucleotide-gated ion channel gene family in wheat and functional analyses of TaCNGC14 and TaCNGC16. Front Plant Sci.

[62] Wang Y, Qiao L, Bai J, Wang P, Duan W, Yuan S, Yuan G, Zhang F, Zhang L, Zhao C (2017). Genome-wide characterization of JASMONATE-ZIM DOMAIN transcription repressors in wheat (*Triticum aestivum* L.). BMC Genomics.

[63] Chauhan H, Khurana N, Agarwal P, Khurana P (2011). Heat shock factors in rice (*Oryza sativa* L.): genome-wide expression analysis during reproductive development and abiotic stress. Mol Gen Genet.

[64] Kotak S, Vierling E, Bäumlein H, Koskulldöring PV (2007). A novel transcriptional Cascade regulating expression of heat stress proteins during seed development of Arabidopsis. Plant Cell.

[65] Jin J, Tian F, Yang DC, Meng YQ, Kong L, Luo J, Gao G (2017). PlantTFDB 4.0: toward a central hub for transcription factors and regulatory interactions in plants. Nucleic Acids Res.

[66] Finn RD, Clements J, Eddy SR (2011). HMMER web server: interactive sequence similarity searching. Nucleic Acids Research.

[67] Letunic I, Bork P (2017). 20 years of the SMART protein domain annotation resource. Nucleic Acids Res.

[68] Kosugi S, Hasebe M, Tomita M, Yanagawa H (2009). Systematic identification of cell cycle-dependent yeast nucleocytoplasmic shuttling proteins by prediction of composite motifs. Proc Natl Acad Sci.

[69] La Cour T, Kiemer L, Mølgaard A, Gupta R, Skriver K, Brunak S (2004). Analysis and prediction of leucine-rich nuclear export signals. Protein Eng Des Sel.

[70] Kotak S, Port M, Ganguli A, Bicker F, Von K-DP (2010). Characterization of C-terminal domains of Arabidopsis heat stress transcription factors (Hsfs) and identification of a new signature combination of plant class a Hsfs with AHA and NES motifs essential for activator function and intracellular localization. Plant J.

[71] Bjellqvist B, Basse B, Olsen E, Celis JE (2010). Reference points for comparisons of two-dimensional maps of proteins from different human cell types defined in a pH scale where isoelectric points correlate with polypeptide compositions. Electrophoresis..

[72] MapGene2Chromosome [http://mg2c.iask.in/mg2c_v2.0/].

[73] Tamura K, Peterson D, Peterson N, Stecher G, Nei M, Kumar S (2011). MEGA5. Molecular evolutionary genetics analysis using maximum likelihood, evolutionary distance, and maximum parsimony methods. Mol Biol Evol.

[74] Hu B, Jin J, Guo AY, Zhang H, Luo J, Gao G (2014). GSDS 2.0. an upgraded gene feature visualization server. Bioinformatics.

[75] Bailey TL, Boden M, Buske FA, Frith M, Grant CE, Clementi L, Ren J, Li WW, Noble WS (2009). MEME SUITE: tools for motif discovery and searching. Nucleic Acids Research.

[76] Zhao LN, Liu ZH, Duan SN, Zhang YY, Guo-Liang LI, Guo XL (2018). Cloning and characterization of heat shock transcription factor gene TaHsfB2d and its regulating role in thermotolerance. Acta Agron Sin.

