# Supplementary figures and images for: Genome-wide identification and abiotic stress-responsive pattern of heat shock transcription factor family in Triticum aestivum L
Source: BMC Genomics. 2019 Apr 1;20:257. doi: 10.1186/s12864-019-5617-1 (PMC6444544; doi:10.1186/s12864-019-5617-1)

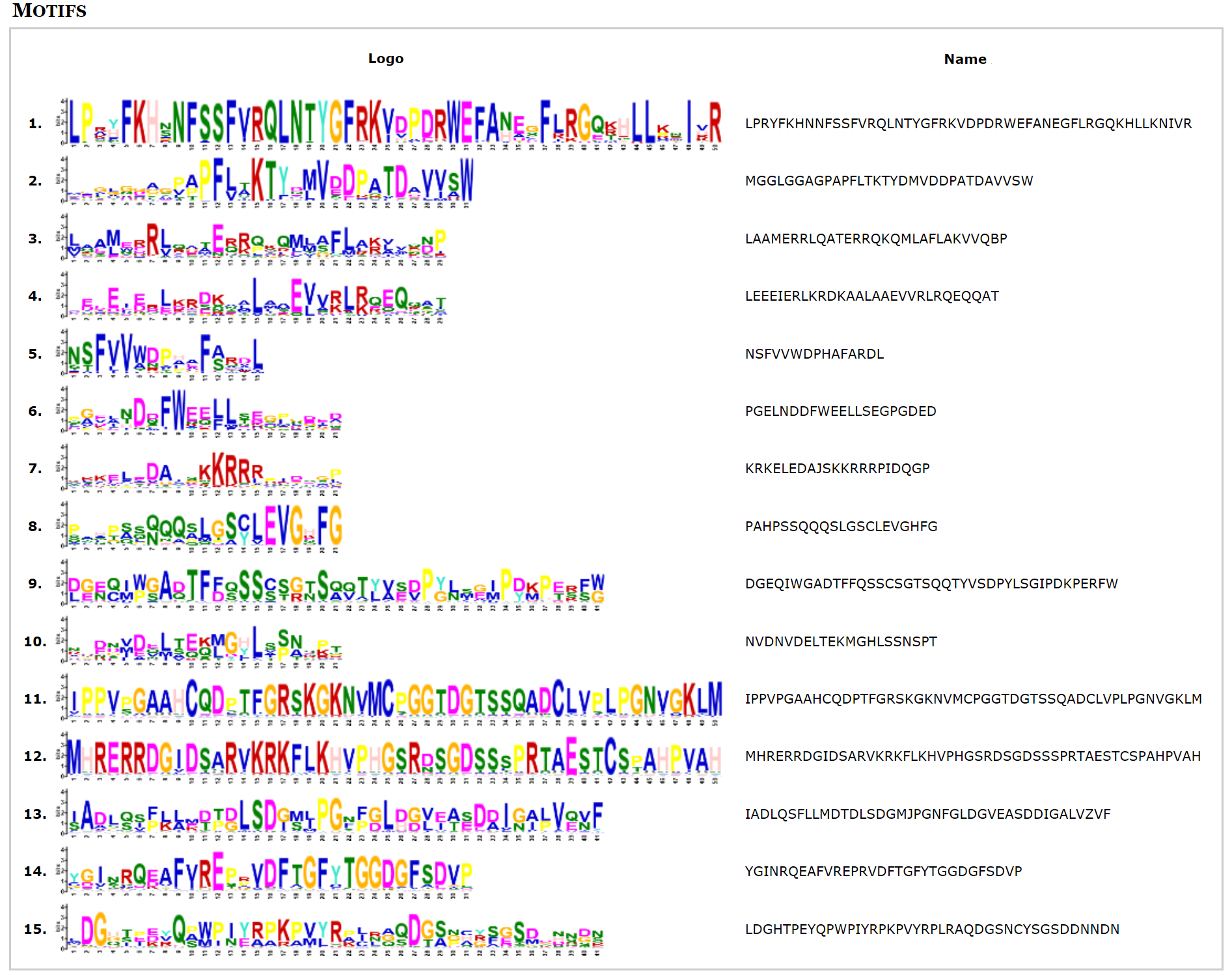

Supplement: Supplementary file 5 — Table S5. Motif sequences identified by MEME tools. (DOC 838 kb) [file 12864_2019_5617_MOESM5_ESM.doc]
